# The single-atom *R*1: a new optimization method to solve crystal structures

**DOI:** 10.1107/S2053273324001554

**Published:** 2024-03-18

**Authors:** Xiaodong Zhang, James P. Donahue

**Affiliations:** a Chemistry Department, Tulane University, 6400 Freret Street, New Orleans, Louisiana 70118, USA; Institute of Crystallography - CNR, Bari, Italy

**Keywords:** structure solution, global minimization, single-atom *R*1, molecular replacement

## Abstract

A crystal structure with *N* atoms in its unit cell can be solved starting from a model with atoms 1 to *j* − 1 being located. To locate the next atom *j*, the method uses a modified definition of the traditional *R*1 factor where its dependencies on the locations of atoms *j* + 1 to *N* are removed. This modified *R*1 is called the single-atom *R*1 (*sR*1), because the locations of atoms 1 to *j* − 1 in *sR*1 are the known parameters, and only the location of atom *j* is unknown. Finding the correct position of atom *j* translates thus into the optimization of the *sR*1 function, with respect to its fractional coordinates, *x*_*j*_, *y*_*j*_, *z_j_*. Using experimental data, it has been verified that an *sR*1 has a hole near each missing atom. Further, it has been verified that an algorithm based on *sR*1, hereby called the *sR*1 method, can solve crystal structures (with up to 156 non-hydrogen atoms in the unit cell). The strategy to carry out this calculation has also been optimized. The main feature of the *sR*1 method is that, starting from a single arbitrarily positioned atom, the structure is gradually revealed. With the user’s help to delete poorly determined parts of the structure, the *sR*1 method can build the model to a high final quality. Thus, *sR*1 is a viable and useful tool for solving crystal structures.

## Abstract 2

A new optimization method based on a new concept of single-atom *R*1 (*sR*1) for solving crystal structures is presented.

## 1. Introduction

Most methods of solving crystal structures in crystallography rely on a Fourier synthesis of an electron-density map. Because the phase angles of the structure factors are not directly measured but are required in the Fourier synthesis, the central theme of solving structures has been recovering the phase angles from the measured reflection intensities. This is generally done in a two-step fashion. The first step is to establish an approximate or partial model for making an initial estimate of the phase angles, and the second step is to iteratively improve the phasing through a dual-space recycling process, which involves reciprocal-space phase refinement and/or real-space electron-density modifications (Weeks *et al.*, 1993 ▸; Miller *et al.*, 1993 ▸; DeTitta *et al.*, 1994 ▸). In the most popular program for solving small structures, *SHELXT* (Sheldrick, 2015 ▸), the starting model is established by the Patterson superposition minimum function (Buerger, 1959 ▸), and the dual-space recycling involves real-space peak picking and reciprocal-space expansion of phases from about 40% of the most reliable phases (Sheldrick *et al.*, 2001 ▸; Schneider & Sheldrick, 2002 ▸). In macromolecular crystallography, the molecular-replacement methods (MR; Hoppe, 1957 ▸; Rossmann & Blow, 1962 ▸; Rossmann, 1972 ▸; Crowther, 1972 ▸; Egert, 1983 ▸; Rossmann & Arnold, 1993 ▸) establish the starting model by optimally placing known models into a target unit cell; in the isomorphous replacement methods (Perutz, 1956 ▸; Kendrew *et al.*, 1958 ▸) the heavy-atom substructure of the derivative is determined from the measured intensity change between the derivative sample and the native sample (the sample before adding heavy atoms); and in single- or multi-wavelength anomalous dispersion/diffraction methods (SAD or MAD; Hendrickson & Teeter, 1981 ▸; Wang, 1985 ▸; Dauter *et al.*, 1999 ▸, 2002 ▸; Dodson, 2003 ▸; Hendrickson, 1991 ▸; Smith, 1998 ▸) the anomalous scatterer substructure is derived from the anomalous differences in the observed reflection intensities. The dual-space recycling step of macromolecular crystallography involves various electron-density-modification techniques: solvent flattening (Wang, 1985 ▸; Leslie, 1988 ▸) or solvent flipping (Abrahams & Leslie, 1996 ▸), and histogram matching and noncrystallographic symmetry averaging (Cowtan & Zhang, 1999 ▸; Terwilliger, 2002 ▸; Brünger *et al.*, 1998 ▸) *etc*.

Though the main routes towards a structure solution in crystallography proceed via phasing, direct routes from the measured reflection intensities to a structure solution have also been explored extensively. The most famous example is the Patterson function, which has been exploited in the model searching step of the molecular-replacement calculations (Hoppe, 1957 ▸; Rossmann & Blow, 1962 ▸; Rossmann, 1972 ▸; Crowther, 1972 ▸; Egert, 1983 ▸; Rossmann & Arnold, 1993 ▸), as well as in various Patterson deconvolution techniques (Richardson & Jacobson, 1987 ▸; Pavelčík, 1988 ▸, 1994 ▸; Burla *et al.*, 2004 ▸, 2006*a* ▸,*b* ▸, 2007 ▸; Caliandro *et al.*, 2008 ▸). The fact that the reflection intensities can be calculated from a trial model by using the atomic scattering factors and the atomic locations (without dealing with the phasing) has also been exploited for solving structures, that is, a structure can be solved via optimization techniques by optimally matching the calculated and the observed reflection intensities. There are a few studies along this line of attack. McCoy *et al.* (2017 ▸) reported that maximization of the log-likelihood gain on intensities (LLGI) had been successful in locating up to ten S atoms in a protein structure with 2525 non-hydrogen atoms. Burla *et al.* (2018 ▸) reported that a diagonal least-squares technique (a simplified version of the least-squares method) had been applied to produce *ab initio* phasing for small crystal structures.

Over a 2-year period, we have been exploring a new optimization method which we term the single-atom *R*1 (*sR*1). The application of *sR*1 for solving crystal structures is, similarly to the LLGI method (McCoy *et al.*, 2017 ▸), a search over a parameter space with the goal of locating atoms or groups of atoms in the unit cell. As this paper is the first formal description of this method, in Section 2 we provide a detailed mathematical derivation of the *sR*1 concept. Though the *sR*1 method has been mathematically derived, its validity rests solely on the test calculations. This is because the derivation is based on mathematical intuition, not on absolute mathematical facts. In Section 3 some basic properties of *sR*1 are explored by calculations with experimental data. In the next few sections, the test calculations for solving structures are described. Along the way, rules for avoiding ghost atoms have been established, and the best strategy for applying *sR*1 to solve crystal structures has been proposed. The success of these test calculations indicates that the algorithm can be used to solve crystal structures. The report ends with a summary and discussion of future directions.

## 2. The single-atom *R*1 (*sR*1) method

A reflection is indexed by *hkl*, and its intensity is proportional to the square of the amplitude of a structure factor. We use the square of the amplitude, *F*_o_^2^(*hkl*), to represent an observed reflection intensity (which has been properly scaled, see below), and the corresponding calculated reflection intensity is *F*_c_^2^(*hkl*), which can be calculated from a model in the following way:

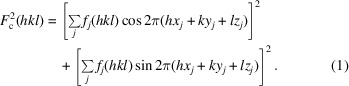

Here atom *j* is located at (*x*_*j*_, *y*_*j*_, *z*_*j*_) and has an atomic scattering factor *f*_*j*_(*hkl*), which is calculated with an isotropic displacement parameter *U* = 0 Å^2^. This choice of *U* is made on purpose, because using *U* > 0 will widen the holes in an *sR*1 map and make the *sR*1 method less precise and less effective in locating missing atoms.

Equation (1) can also be expressed in the following form:

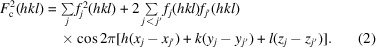

In this expression the first sum is positive and depends only on the number and types, not on the locations, of the atoms. The second sum depends on the relative location of pairs of atoms and includes positive and negative values because the cosine function has values from −1 to +1. These contributions largely cancel each other when the intensities of all reflections are added together and, so, the following approximation can be reached:

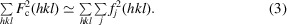

This approximation suggests that the observed intensities *F*_o_^2^(*hkl*) should be scaled such that their sum equals Σ_*hkl*_Σ_*j*_ *f*_*j*_^2^(*hkl*). This scaling rule will be applied in the *sR*1 method.

We use the traditional *R*_1_ to evaluate the mismatch between the calculated and the observed intensities:

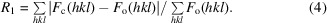




*R*_1_ can be considered a function of the 3*N* atomic coordinates. Thus, in principle, an atomic model can be solved by globally minimizing *R*_1_ while simultaneously adjusting the locations of all *N* atoms in the unit cell. Of course, such a brute-force calculation is too overwhelming for the current computing technology. Therefore, we seek a stepwise algorithm, in which each step only determines the location of one atom.

The first atom can easily be located. As seen in equation (2), *F*_c_^2^(*hkl*) only depends on the relative positions of the pairs of atoms. So, the absolute location of the whole structure is immaterial, and the first atom can be put at any location. For convenience of programming, we choose (0.3, 0.3, 0.3) for the location of the first atom, although any other location can serve this purpose. As to which atom should be selected as the first atom, the choice falls on the heaviest atom, so that the first atom accounts for as much scattering power as possible.

Let us next turn our attention to the following steps. Here we assume that the locations of atoms 1 to *j* − 1 have been determined. To determine the location of atom *j*, we use the *R*1 as defined by equation (4), in which only *F*_c_(*hkl*) depends on the locations of atoms. This dependency can be seen in equation (2), where the locations of all *N* atoms are involved. As we already know the locations of atoms 1 to *j* − 1, we need not worry about these locations. The locations of the remaining atoms *j* to *N* are still unknown. But we only want to find the location of atom *j* at this step, so we should remove any dependency of *F*_c_^2^(*hkl*) on the locations of atoms *j* + 1 to *N*. By deleting all terms that are dependent on the locations of atoms *j* + 1 to *N* from equation (2), the resulting approximate equation can be manipulated into the following form:

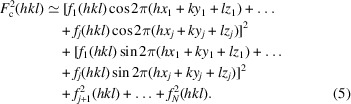

Note that one may be tempted to derive this equation by deleting all terms that are dependent on the locations of atoms *j* + 1 to *N* from equation (1). In that case, one reaches an equation like equation (5) but without the tail 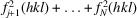 . We have tested the method with such a variant but found that the results are much worse than when using equation (5). Of course, this is understandable, because retaining 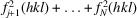 makes the calculation of *R*1 more accurate.

Now we can give a formal definition of the single-atom *R*1 (*sR*1): when *F*_o_^2^(*hkl*) are scaled such that their sum equals Σ_*hkl*_Σ_*j*_ *f*_*j*_^2^(*hkl*), when *F*_c_^2^(*hkl*) are calculated by the approximation of equation (5), and when the coordinates of atoms 1 to *j* − 1 are the known parameters, the calculated traditional *R*1 depends on the unknown coordinates of atom *j* only and, so, it is called the single-atom *R*1 (*sR*1).

In *sR*1 the locations of atoms 1 to *j* − 1 appear as known parameters, while the only unknown parameters are the coordinates of atom *j*: (*x*_*j*_, *y*_*j*_, *z*_*j*_). So, atom *j* acts as a probe atom. Up to now, atoms 1 to *j* − 1 have already been put into the model, and atoms *j* to *N* are missing from the model. When we move the probe atom *j* around, *sR*1 will change. Intuition tells us that when the probe moves over the location of a missing atom, *sR*1 will decrease, because now the probe accounts for the diffraction power of that location, though it could be under- or overestimating it. When the probe starts to move away from that location, *sR*1 will increase as the probe is entering the regions where no diffraction power needs to be accounted for. So, we predict that there is an *sR*1 hole at the location of each missing atom.

One can ask: among all the missing atoms from *j* to *N*, which atom should be selected as the probe? Again, intuition tells us that we should select the heaviest atom, which potentially yields the deepest *sR*1 holes. At least, if we choose the heaviest missing atom as the probe, when the probe moves over the location of the heaviest missing atom, we reach the deepest *sR*1 hole. So, in such an arrangement, in each step, the deepest *sR*1 hole locates the heaviest missing atom. And this completes the derivation of the *sR*1 algorithm.

The *sR*1 algorithm can be carried out in two ways. In the first, one finds all local minima (holes) of the *sR*1 function, making sure to discard those not corresponding to coordinates within the unit cell. Later on, the lowest among such minima is selected as the correct location for the atom *j*. In the second, one directly searches for the global minimum point and assigns its location to atom *j*, because the minimum point of the deepest *sR*1 hole must be the global minimum point. As global minimization is arguably more straightforward to program, we have initially used this approach. However, as shown later, and counter to our intuition, the first approach turns out to be more efficient.

In Section 3 we do some calculations to verify that there is an *sR*1 hole nearby each missing atom. The success of the trial calculations in the few sections after Section 3 serves as the verification of the other prediction, namely, the deepest *sR*1 hole locates the heaviest missing atom.

In the current implementation of this *sR*1 method, all calculations are performed in the minimal *P*1 space group, and any centering is also ignored. This strategy has been proved to be robust in some other programs (Sheldrick & Gould, 1995 ▸; Burla *et al.*, 2000 ▸; Caliandro *et al.*, 2007 ▸; Sheldrick, 2015 ▸). The reflection data are expanded to *P*1 by complementing any missing symmetry equivalents, and Friedel pairs are also fully expanded. Duplicate reflections are merged by taking a simple (non-weighted) average. The calculation result naturally takes care of any existing crystallographic symmetry (including centering), which can later be recovered by examining the resulting cell content. However, this extra step is not fully performed for the examples in this report: only the space group is extracted using the *PLATON* (Spek, 2020 ▸) program to analyze the final model, but shifting the cell origin to match the convention and extracting the asymmetric part of the structure are not performed.

## 3. Basic properties of *sR*1

Three samples, samples 1, 2 and 3, are selected and studied in detail in this report. Table 1 ▸ gives the crystallographic information of these samples.

**Figure 1:**
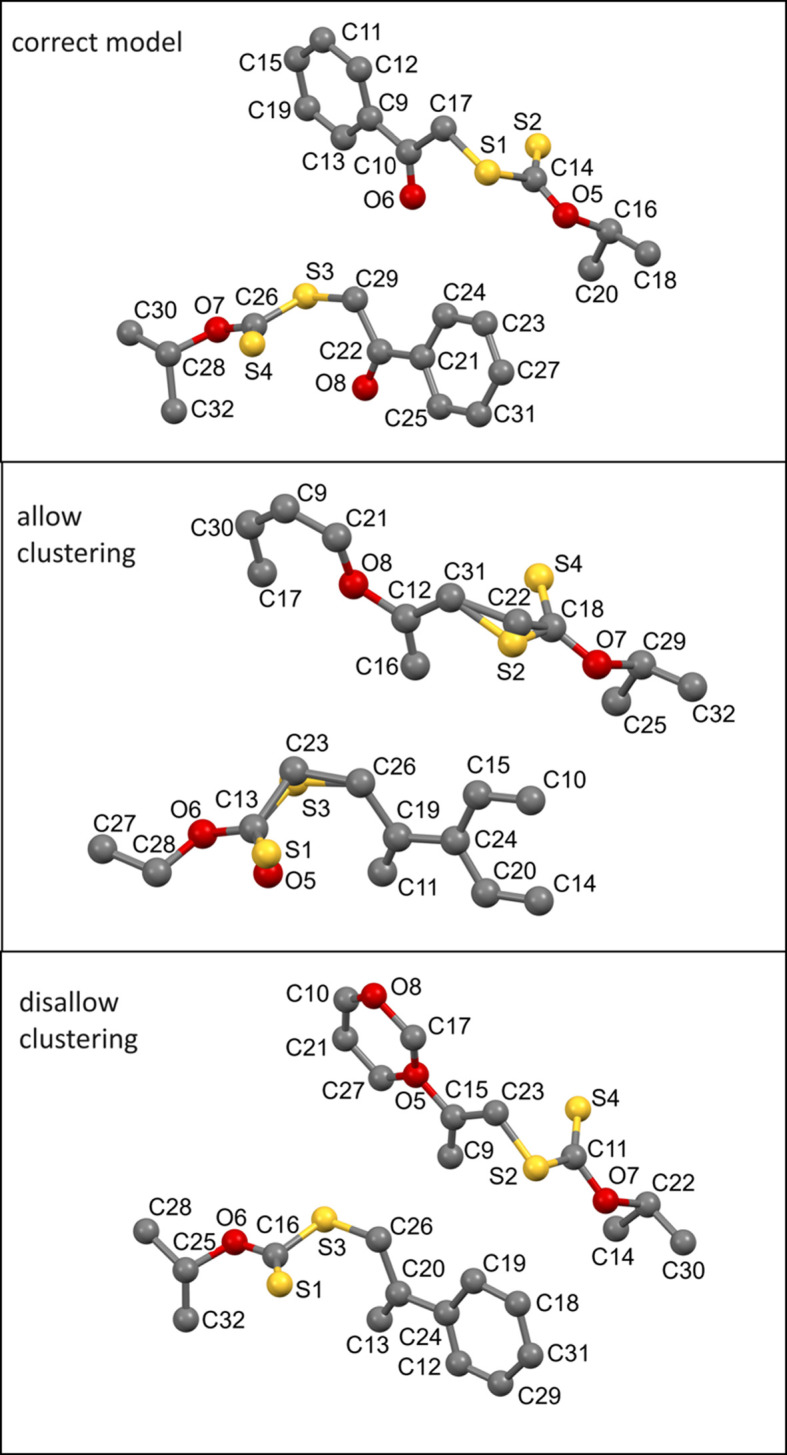
The models of sample 1. Top panel: the correct model. Middle panel: the resulting model via global minimization calculation that allows clustering ghost atoms. Bottom panel: the resulting model via global minimization calculation that excludes clustering ghost atoms.

Sample 1 is used to reveal the basic properties of *sR*1. The correct model of sample 1 is shown in the top panel of Fig. 1 ▸.

Note that in the correct model of sample 1, as shown in the top panel of Fig. 1 ▸, the atoms are numbered from the heaviest to the lightest: S1 to S4, O5 to O8, and C9 to C32. Different *sR*1’s are defined, depending on which atoms are known. Three *sR*1’s will be studied: *sR*1(S2) for which only S1 is the known atom; *sR*1(O7) for which S1 to S4 and O5 to O6 are the known atoms; and *sR*1(C20) for which S1 to S4, O5 to O8, and C9 to C19 are the known atoms. All holes of each *sR*1 within the unit cell are discovered, and each missing atom is assigned an *sR*1 hole that is closest to it. The distance between a missing atom and the minimum point of its assigned *sR*1 hole is listed in Table 2 ▸. The listed data in Table 2 ▸ show that *sR*1(S2) can locate the missing atoms within 0.00 to 0.41 Å, *sR*1(O7) can locate the missing atoms within 0.00 to 0.19 Å, and *sR*1(C20) can locate the missing atoms within 0.03 to 0.13 Å. Therefore, in general, when the model becomes more complete, an *sR*1 can locate the missing atoms more accurately. But even the worst accuracy 0.41 Å is smaller than 0.5 Å, so, in general, global minimization of an *sR*1 can locate each missing atom within 0.5 Å.

**Figure 2:**
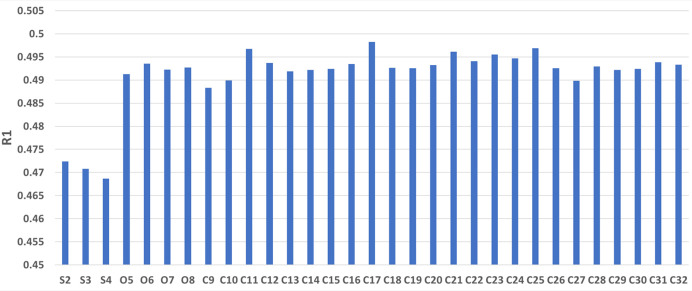
The *R*1 value at the minimum point of each *sR*1(S2) hole that is next to a missing atom.

Fig. 2 ▸ shows the *R*1 values of the minimum point of the *sR*1(S2) holes that are neighbors of each missing atom. The data in Fig. 2 ▸ indicate that the heavier missing atoms S2 to S4 have much deeper *sR*1 holes than the lighter atoms O5 to O8 and C9 to C32.

The profiles of an *sR*1 hole can also be calculated (details are provided in the supporting information). In a typical *sR*1 profile along a straight line passing through the minimum point of an *sR*1 hole, the local minimum point of the hole is sandwiched between two neighboring local maximum points, and the distance between these two maximum points is larger than 1.2 Å. This indicates that we may choose 0.4 Å step size for setting up a coarse grid for performing the global minimization of *sR*1’s, as well as for locating the *sR*1 holes.

## 4. Avoiding clustering ghost atoms

As predicted in Section 2, the global minimization of *sR*1’s can sequentially locate the atoms of an unsolved structure. Taking sample 1 as an example, at the start, a single atom S1 is placed at (0.3, 0.3, 0.3) and this provides a known atom for defining *sR*1(S2). Next, S2 is located by globally minimizing *sR*1(S2). To perform this global minimization, a coarse grid is set up within the whole cell with a step size of 0.4 Å. The *sR*1 values are calculated on all grid points and the grid point with the smallest *sR*1 value marks the global minimum point. The precision of locating this minimum point is improved to 0.001 Å by repeatedly halving the step size locally (more technical details are given in the supporting information). At this point, both S1 and S2 are known, so *sR*1(S3) can be defined and globally minimized to locate S3. This step is repeated. Finally, S1 to S4, O5 to O8 and C9 to C31 are all known, and *sR*1(C32) can be defined and globally minimized to locate the last atom C32. The final resulting model of this procedure is shown in the middle panel of Fig. 1 ▸. This resulting model has the following problems: O5 is too close to S1 (only 0.55 Å distance), C22 is too close to S2 (only 0.47 Å distance) and C23 is too close to S3 (only 0.43 Å distance). Atoms like O5, C22 and C23 are called the clustering ghost atoms because they are chemically incorrect. To avoid these clustering ghost atoms, the following rule should be enforced: when a candidate location is too close to a known atom (within an exclusion radius that depends on the type of known atom), this location should be disqualified as a candidate for global minimization. If the known atom is one of the heavy atoms like I, Mo, Pd and Se *etc*., the exclusion radius is chosen at 2.2 Å, while for all other atoms 1.2 Å is chosen. These choices are based on trial and error. With this rule, the global minimization steps lead to the resulting model shown in the bottom panel of Fig. 1 ▸. The model has only the following minor problems: O5 should be assigned as C5, O8 should be C8, C9 should be O9, and C13 should be O13. These minor problems can be corrected in the structure refinement step. (Interestingly, if O5, O8, C9 and C13 are deleted and the model is rebuilt with another batch of global minimization steps, these atoms will be assigned correctly.)

In the *sR*1 method, the atomic scattering factors are calculated with the isotropic displacement parameter *U* = 0. Obviously, this atomic model does not match the real electron density of an atom. It accounts for the electron density at the central region of a real atom. Nearby this central region there is some unaccounted-for electron density, which may fool the probe atom and make an *sR*1 hole deep enough to produce a ghost atom, especially when the already located atom is a heavy one. This is the plausible formation mechanism of the clustering ghost atoms.

## 5. Preventing ghost atoms at the center of completely or partially formed benzene rings

**Figure 3:**
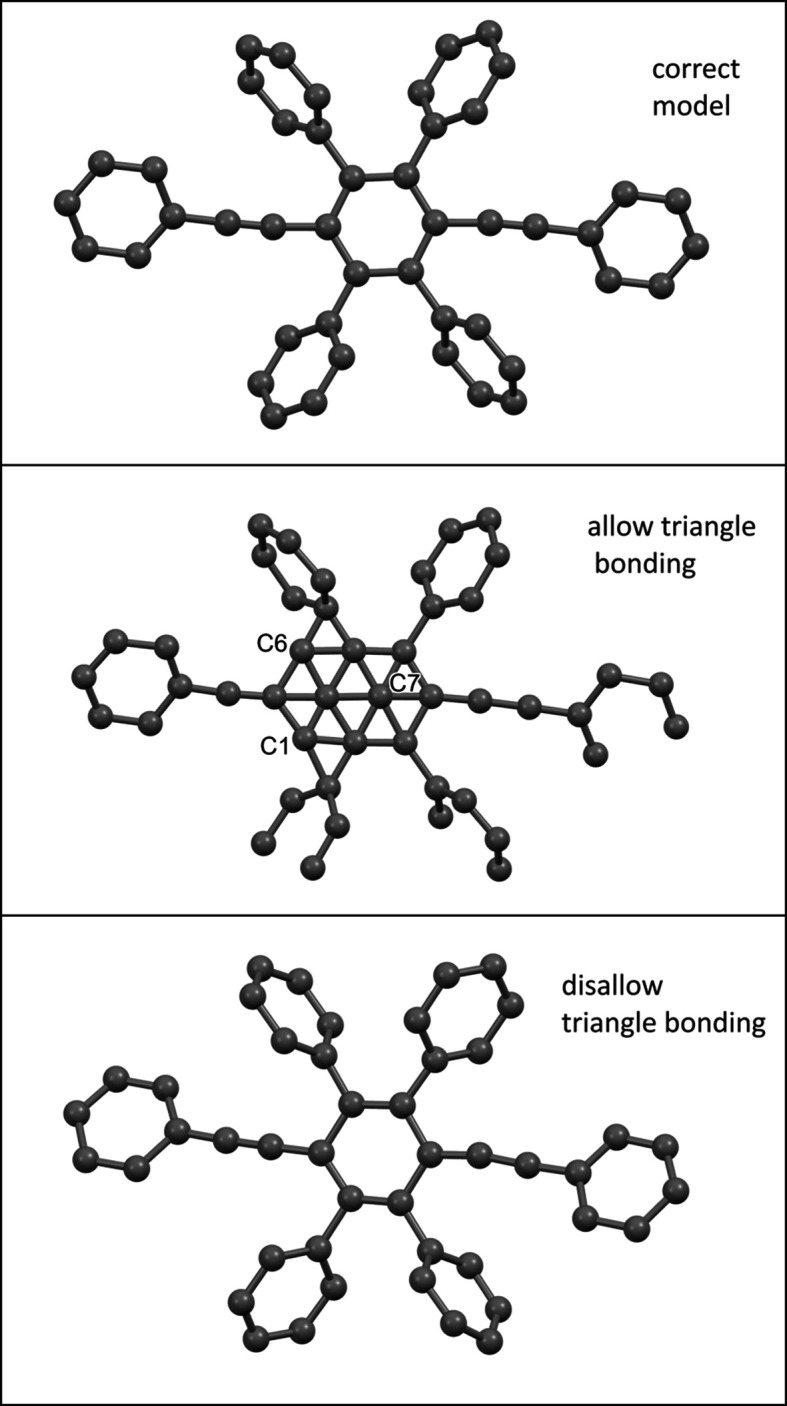
Models of sample 2. Top panel: the correct model. Middle panel: the resulting model by applying global minimization of *sR*1’s with the rule excluding clustering ghost atoms (but triangular bonding is not excluded). Bottom panel: the resulting model when both clustering ghost atoms and triangular bonding are excluded.

The correct model of sample 2 is shown in the top panel of Fig. 3 ▸. This structure has several benzene rings. Applying the global minimization of *sR*1’s with the rule excluding clustering ghost atoms to this sample, the resulting model is shown in the middle panel of Fig. 3 ▸. This resulting model has a new type of ghost atom: C7 is a ghost atom located at the center of a benzene ring, and C1 and C6 are two ghost atoms at the centers of two partially formed benzene rings (each with only four atoms, short of the six atoms for a complete benzene ring). These ghost atoms form triangular bonds with their hosting (complete or partial) benzene rings. Therefore, these ghost atoms can be prevented if a rule excluding triangular bonding is enforced: given that atoms *A* and *B* have already been located and *L* is a candidate location, if *L* can form triangular bonding with *A* and *B* with all three bonds shorter than 1.6 Å, then *L* should be disqualified as a candidate. Using this rule to re-do the global minimization steps, the resulting model, as shown in the bottom panel of Fig. 3 ▸, will be the same as the correct model.

There is one catch in enforcing the rule excluding triangular bonding: again, consider the atoms *A* and *B*, and the candidate location *L*. What if one of *A* and *B* is a ghost atom, while *L* is a correct location, and the three make triangular bonding? In this instance, enforcing the rule will exclude this correct location. One possible strategy is to abandon this rule, and after finishing the model, manually delete the ghost atoms and then re-do the global minimization steps to complete the model again. However, this approach would be frustrating: the deleted ghost atoms, with triangular bonding allowed, will keep coming back. A better strategy is to enforce the rule excluding triangular bonding, even though this rule may occasionally lead to mistakes. The mistakes can be later corrected manually by deleting the ghost atoms. After deleting the ghost atoms, the global minimization steps are repeated to complete the model again. This time, because the calculation is now based on a more complete model, the result is more accurate such that the correct locations win the competition and get adopted before the ghost locations (even though some ghost locations have a chance of being examined, they are rejected by the rule excluding triangular bonding).

For structures containing benzene rings, when the probe atom moves over the center of a benzene ring, it simultaneously accounts for part of the diffraction power of all six atoms in the ring; in this way, an *sR*1 hole forms, and when deep enough, a ghost atom will be created. This is the plausible formation mechanism of a ghost atom at the center of a benzene ring.

## 6. Using *sR*1 holes to predict the possible locations of all missing atoms

**Figure 4:**
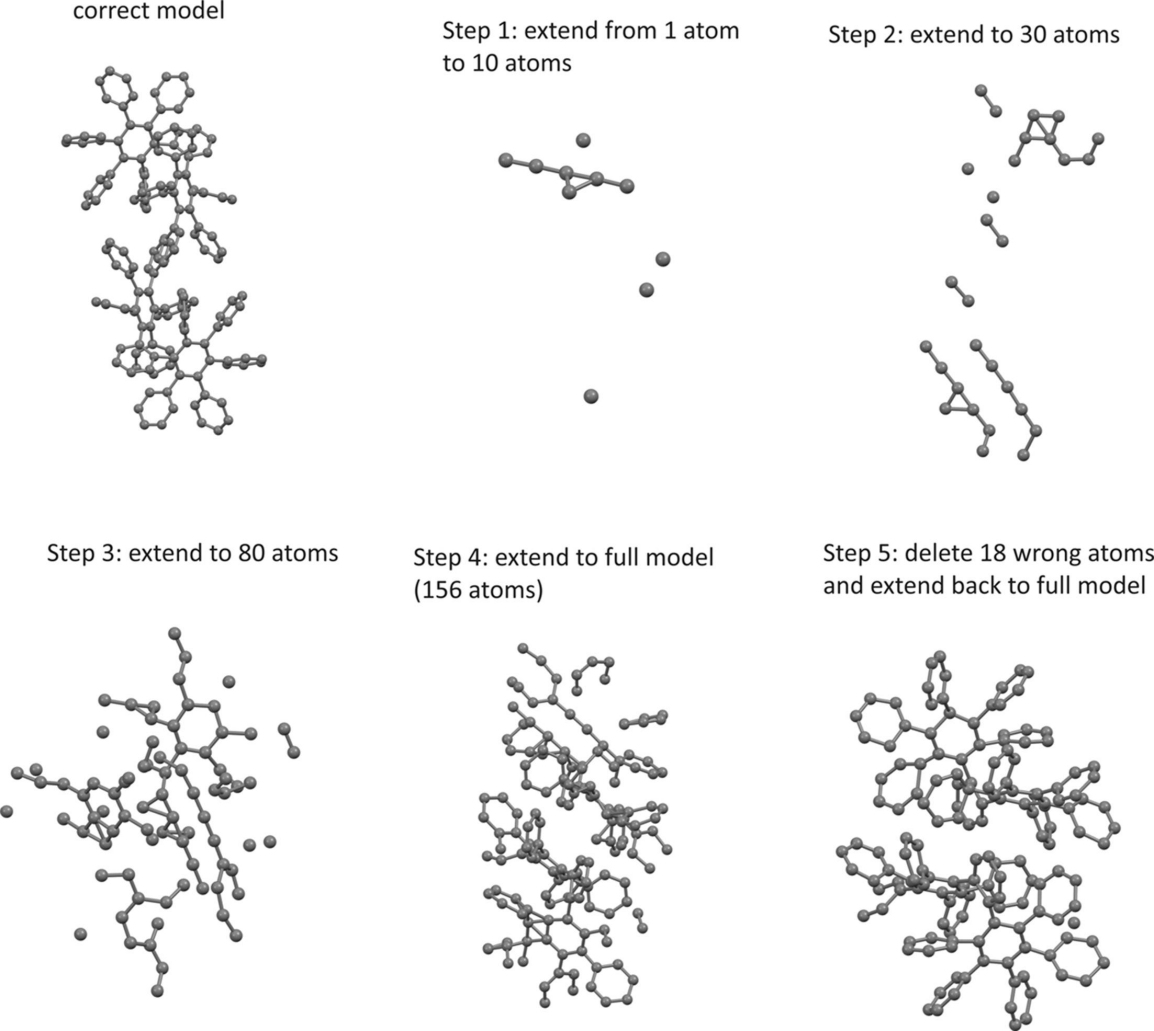
Correct model of sample 3 and the steps in solving this structure (using batches of global minimization steps that are started by predicting the locations of the missing atoms by *sR*1 holes).

Because an *sR*1 map has holes at the locations of the missing atoms, the set of all holes encompasses all locations of the missing atoms. Therefore, the searching range of the global minimization of an *sR*1 can be limited to these holes only. This strategy can greatly shorten the calculation time of these global minimization steps. The benefit of this strategy may not be apparent when solving a small structure like sample 1, but is enormous when a large structure like sample 3 is to be solved. The correct model of sample 3 is shown in the first panel of Fig. 4 ▸. It has 156 C atoms in its unit cell. The steps of solving the structure by this new strategy are also shown in Fig. 4 ▸. The calculation starts by placing a single C atom at (0.3, 0.3, 0.3). In step 1, first, the possible locations of all missing C atoms are predicted by the holes of the *sR*1 map based on the single known C atom. The holes are coarsely discovered via a grid of 0.4 Å step size: a grid point marks a hole if it has smaller *R*1 than all six neighboring grid points. The precision of locating a hole is improved by repeatedly halving the step size locally (more technical details are given in the supporting information). Since the holes will only serve to estimate the locations of the true minimum points of an *sR*1, the final precision will be further refined to 0.001 Å after identifying that a candidate point is close to the true global minimum point. Therefore, the initial precision does not need to be high, in order to save calculation time; but it should not be too low either (to avoid selecting a hole that is not the deepest). As a compromise, we choose 0.1 Å as the initial precision. Typically, there are 2000 to 5000 holes in an *sR*1 map of sample 3. Taking the deepest 780 (five times the total 156 C atoms in the cell) holes is sufficient because the holes of the missing atoms are deeper than the noise. After predicting the possible locations of the missing atoms, we do a batch of global minimization steps (in each step we only search over these 780 candidate locations) to extend the model to ten atoms. In steps 2, 3 and 4 we do the same thing and extend the model to 30, 80 and the full 156 atoms, respectively, to reach an intermediate full model. This intermediate full model is quite complete, and its 18 ghost atoms are identified and deleted. In step 5, after deleting these ghost atoms and again predicting the locations of the missing atoms, we do a final batch of global minimization steps to extend back to the full model again. This final full model has only one atom misplaced. The whole process of solving the structure by this new strategy takes about 1 h to complete. In comparison, if the calculation is done with the complete set of 0.4 Å step size grid points (a total of 52 080 points) of the whole cell as candidate locations for performing global minimization, the full model is finished in about 18 h. Even worse, the resulting model only has 73 out of 156 atoms located within 0.5 Å, and out of all 156 atoms only about 20 atoms form recognizable fragments. Therefore, manually deleting all atoms that are not guaranteed to be correct and repeating the calculation for a few cycles to reach the final correct model will cause it to take many additional hours to complete.

It is somewhat surprising that the quality of the resulting model when the full 52 080 grid points are taken as candidates is worse than when only 780 selected points are used in the global minimization steps. At first glance, using all grid points should guarantee that the minimization is carried out truly ‘globally’, so its result should be better, or at least the same. One should realize that the problem comes from enforcing the rule excluding clustering ghost atoms. This rule cuts ball-shaped holes in the unit cell. These holes are centered at the known atoms. When the true global minimization is carried out, sometimes the global minimum is realized at the edge of these holes. But this is not a true local minimum point and leads to a ghost atom. On the other hand, the selected 780 points are all true local minimum points, and in using them as candidates, even though the same rule is enforced, no new artificial ghost atoms are created. Thus, the new strategy unexpectedly brings the additional benefit of generating higher-quality models.

One may notice that, after putting the first C atom at (0.3, 0.3, 0.3), one can predict the possible locations of all the missing atoms by using the *sR*1 holes. One may be tempted to immediately extend the model from one atom to the full 156 atoms. However, the result of this rushed calculation is not good: only 32 out of 156 atoms are located within 0.5 Å, and hardly any fragment can be identified as a correct fragment, thus excluding the possibility of applying manual editing. This type of rushed calculation fails because of the low accuracy of using the *sR*1 holes to represent the locations of the missing atoms when the model is still very incomplete. Therefore, the correct strategy, as carried out above, is to only extend by a few atoms at the start. Later, when the model becomes more complete, it is possible to extend with more atoms at each batch of the global minimization steps. The sequence of extending from one to ten to 30 to 80 to 156 atoms has been proven to be a good strategy for solving the structure of sample 3.

## 7. Effect of inaccurate estimates of the cell content on the structure solutions

Quite often in crystallography the initial estimate of a cell content is off (*i.e.* inaccurate). With an initial estimate of the cell content being off by about 20% (more or less), we have tested samples 1 to 3 with the *sR*1 calculations. Details of these tests are presented in the supporting information. We have found that the *sR*1 method still works when an initial estimate of the cell content is off. When the cell content is underestimated, the resulting model is correct except that some atoms are missing, and it is clear which atoms are missing. When the cell content is overestimated, the correct model can be determined but with additional spurious atoms, which should be deleted. In either case, a user can eventually determine the correct content and the correct structure. If analyzed mathematically, when the cell content is off, the scaling of *F*_o_^2^(*hkl*) will be off. The calculation of *F*_c_^2^(*hkl*) will also be off, and in the end the absolute value of *R*1 will be off. However, the probe atom can still correctly detect *sR*1 holes because such detection depends on the variation of *R*1 with the location of the probe. It is understandable that when the probe moves over the location of a missing atom, *R*1 will still dip, even though the absolute value of *R*1 is off. For this reason, the *sR*1 method can correctly locate the missing atoms when the cell content has been estimated incorrectly.

When the initial estimate of the cell content is off, the scaling of *F*_o_^2^(*hkl*) will be accordingly off, but the *sR*1 method will still work correctly. One may wonder: what will happen if the initial estimate of the cell content is correct, but the scaling of *F*_o_^2^(*hkl*) itself is off? For this, we have tested (for details see the supporting information) and found that the *sR*1 method will still work correctly even if the scaling is doubled or is halved. Though the overall calculation is still correct, during the calculation there are more ghost atoms that need to be deleted. In general, the best scaling is the one that we have been using, namely, the intensities should be scaled such that their sum equals Σ_*hkl*_Σ_*j*_ *f*_*j*_^2^(*hkl*). The reason that the *sR*1 method works correctly even when the scaling is off is the same: the *sR*1 method only depends on its ability to detect *sR*1 holes, and when the scaling is off, *sR*1 still correctly dips when the probe moves over the location of a missing atom.

## 8. Effect of truncating reflection data resolution to 1 Å on the structure solution strategies

The raw reflection data are used in all the above calculations. The raw data resolutions are: sample 1, 10.4–0.84 Å; sample 2, 12.39–0.73 Å; sample 3, 15.02–0.77 Å.

**Figure 5:**
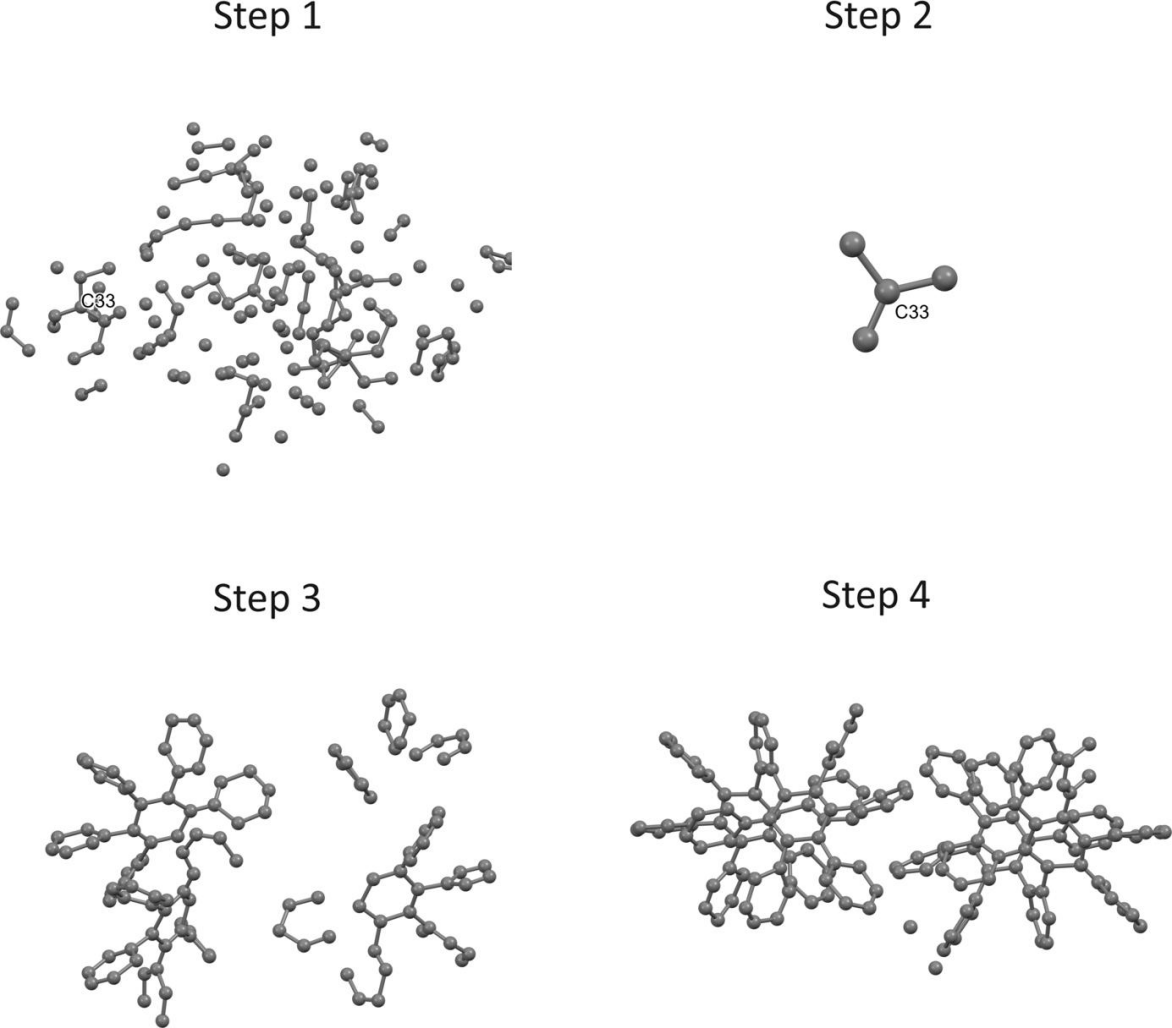
One attempt to solve the structure of sample 3 after truncating the data resolution to 1 Å (using batches of global minimization steps that are started by predicting the locations of the missing atoms by the *sR*1 holes). Step 1: starting from a single C atom, extend the model to ten, to 30, to 80 and to 156 atoms. Step 2: select a small fragment. Step 3: extend the model to ten, to 30, to 80 and to 156 atoms, and delete the ghost atoms. Step 4: extend the model to 156 atoms.

If the data resolution is truncated to 1 Å, using batches of global minimization steps that are started by predicting the locations of the missing atoms by the *sR*1 holes, the structures of samples 1 and 2 can be solved, although it does require extra cycles of deleting ghost atoms and re-extending back to full models. However, handling sample 3 is tricky. As indicated by the first step shown in Fig. 5 ▸, doing a cycle from a single atom to the full model as normal, starting from a single C atom at (0.3, 0.3, 0.3), then extending to ten, to 30, to 80 and to 156 atoms, the resulting model is mostly unrecognizable. To resolve this dilemma, one must try to identify, or guess, a likely correct small fragment of the structure. Once such a fragment is identified, all the other atoms can be deleted. If this fragment is a correct fragment, or is nearly correct, the *sR*1 holes derived from this fragment can represent the missing atoms more accurately than those derived from a single starting atom. So, continuing to extend the model from this fragment will more likely lead to the correct solution. As shown in the second step of Fig. 5 ▸, a small fragment of four C atoms is selected; this fragment’s central atom is labeled as C33 in both step 1 and step 2. This small fragment is suggested to be a correct fragment. Indeed, as seen in steps 3 and 4 of Fig. 5 ▸, by extending from this fragment, the correct model can be induced and then improved.

## 9. Extending the *sR*1 concept to a more general concept of partial structure *R*1 (*pR*1)

**Figure 6:**
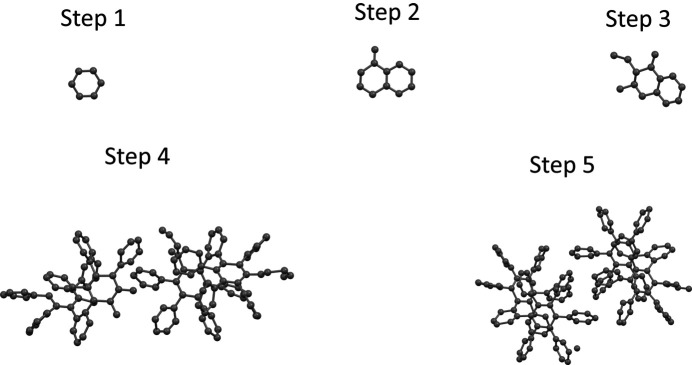
Another attempt to solve the structure of sample 3 after truncating the data resolution to 1 Å. Step 1: place a single benzene ring with its center at (0.3, 0.3, 0.3) and with its orientation determined by the deepest hole in a *pR*1 map of a three-dimensional orientation space. Step 2: using *sR*1, extend the model to 30 and to 80 atoms, and keep a recognizable fragment. Step 3: repeat step 2. Step 4: using *sR*1, extend the model to 30, to 80 and to 156 atoms, and delete the ghost atoms. Step 5: using *sR*1, extend the model to 156 atoms.

For the difficult case of sample 3 with the truncated resolution, one strategy to jumpstart the global minimization steps is to use a single benzene ring that is correctly oriented. To correctly orient a fragment like a benzene ring, it is necessary to extend the *sR*1 concept to a more general concept called the partial structure *R*1 (*pR*1). The same equation (5) is used to define *pR*1. When defining *sR*1, among the atoms 1 to *j*, only *j* is the single missing atom. For defining *pR*1, there are multiple missing atoms: 1 to *i* − 1 are the known atoms, while *i* to *j* are the missing atoms. The missing atoms *i* to *j* form a fragment, or a partial structure, or a partial model. The structure of this fragment is known. For example, the fragment could be a benzene ring with a bond length of 1.39 Å. If the fragment contains a single atom, *pR*1 turns into *sR*1. Just like an *sR*1 can locate a single atom by the deepest hole in an *sR*1 map of a three-dimensional location space, in general, a *pR*1 can determine the orientation and location of a missing fragment by the deepest hole in a *pR*1 map of a six-dimensional orientation-location space (or five-dimensional if the fragment is linear). In a special case, when the fragment is free-standing, that is, there are no known atoms, then the location of this free-standing fragment is immaterial, and the correct orientation of this fragment can be determined by the deepest hole of a *pR*1 map of a three- or two-dimensional orientation space. To locate the deepest *pR*1 hole in an orientation space, a coarse grid of step size 5° in rotation angles is set up. The value of *pR*1 is calculated over all grid points, and the grid point with the smallest *pR*1 marks the global minimum point (the same as the deepest hole). The precision of locating this minimum point is further refined by halving the step size five times locally (more technical details are given in the supporting information). Applying this method to sample 3, a single benzene ring is placed with its center at (0.3, 0.3, 0.3), and its correct orientation is determined by the deepest hole of the *pR*1 map of a free-standing benzene. This correctly oriented benzene ring is shown in step 1 of Fig. 6 ▸. Starting from this correctly oriented benzene ring, applying batches of global minimization of *sR*1’s, combined with manual deletion of bad or uncertain atoms, as shown in steps 2 to 5 in Fig. 6 ▸, one eventually reaches the correct model of sample 3.

## 10. General strategies of using *sR*1 to solve crystal structures

The *sR*1 map has holes at the locations of missing atoms, and the deepest hole locates the heaviest missing atom (after avoiding clustering ghost atoms and excluding triangular bonding). As the holes of an *sR*1 map encompass all missing atoms, when a new *sR*1 is defined by including the newly located atom as a known atom, it is not necessary to re-discover a new set of *sR*1 holes. Instead, the deepest hole of the new *sR*1 map can be discovered by testing the locations of the old holes. However, the *sR*1 holes are inaccurate representations of the locations of the missing atoms when the model is very incomplete. They become more accurate representations when the model becomes more complete. So, at the start, after finding the *sR*1 holes, the first batch of using the deepest *sR*1 holes to extend the model should only extend the model by a few atoms; at later stages the number of atoms being extended at each batch of such a calculation can increase quickly. Finally, the resulting model should always be examined for chemical soundness, and the atoms that do not make chemical sense should be deleted. To minimize propagation of mistakes, it is better to correct any identifiable mistakes as early as possible.

After deleting the ghost atoms, the calculation is resumed. As a matter of fact, *sR*1 can also be used to improve the quality of the model. Any deformed part of the model, for example, a distorted benzene ring, can be deleted and rebuilt to better quality. Sometimes, one may meet difficult cases like that of sample 3 after truncating the data resolution to 1 Å. In these difficult cases, the resulting model is mostly unrecognizable. In such cases, it is necessary to look for a small fragment which looks more likely to be correct. Alternatively, one may place a known fragment with its local origin (for more technical details about the local origin of a fragment, see the supporting information) at (0.3, 0.3, 0.3) and use the deepest hole in a *pR*1 map of a free-standing fragment to determine its correct orientation. Extending the model from a correct fragment leads to more recognizable fragments. By retaining these recognizable fragments and extending from them, one eventually reaches the full structure.

**Figure 7:**
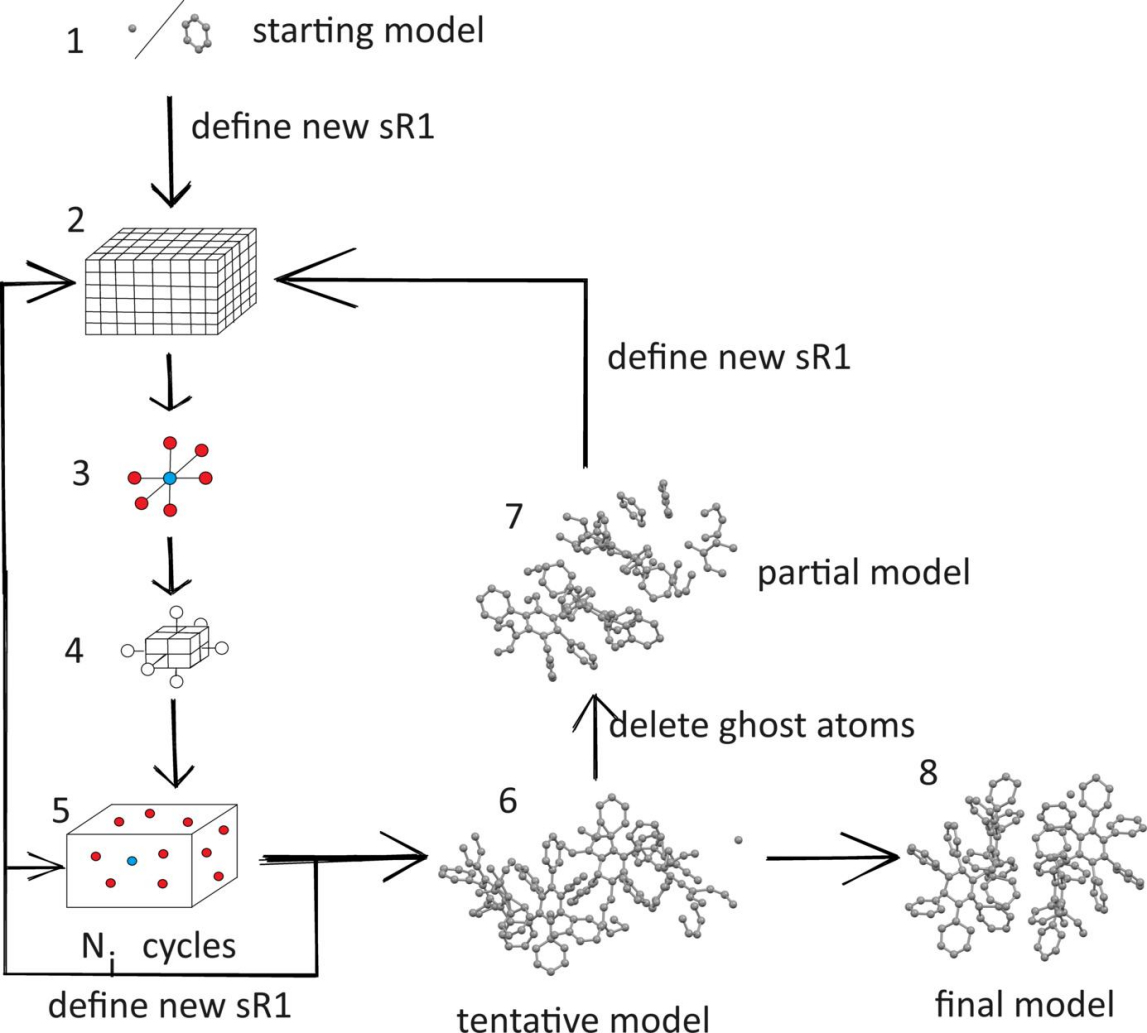
A general flowchart of applying the *sR*1 method for solving an unknown structure. Step 1: the starting model, which is either a single atom that is arbitrarily positioned, or a known fragment that is also arbitrarily positioned but is correctly oriented by *pR*1 of a free-standing fragment. After defining a new *sR*1 the calculation moves on to step 2: set up a grid within the cell with step size 0.4 Å. Note that whenever defining a new *sR*1 the known model up to that point is used. Step 3: discover *sR*1 holes. A grid point (colored blue) with an *sR*1 value smaller than all its six neighbors (colored red) marks an *sR*1 hole. Step 4: use a local grid with halved step size to refine the location of the *sR*1 holes. The local grid point with the lowest *sR*1 is accepted as the improved location of the hole. After repeating step 4 one more time, the 5*N* of the deepest holes are adopted as the candidate locations; here *N* is the number of atoms expected in the unit cell. Step 5: locate atom *j*. First, the candidate locations are filtered by applying rules excluding ghost atoms. The filtered candidate locations are shown as colored balls. Calculate *sR*1 over these candidate locations and assign the one (colored blue) with the smallest *sR*1 to atom *j*. The precision of this location is refined to 0.001 Å. After defining the new *sR*1 repeat step 5. In total, step 5 is executed *N_i_* times. Note that *N_i_* represents one number from a set of *N*_1_, *N*_2_, *N*_3_, …, and this set constitutes a calculation strategy. For example, for sample 3, the set of *N_i_*’s is 9, 20, 50, 76. Note that *N*_1_ + *N*_2_ + *N*_3_ + … = *N* − *N*_0_, where *N*_0_ is the number of atoms in the starting model. After finishing *N_i_* cycles of step 5, with a newly defined *sR*1, the calculation goes back to steps 2, 3, 4, then executes the next *N_i_* cycles of step 5. Repeat this until all *N_i_*’s in the set *N*_1_, *N*_2_, *N*_3_, …, are finished. At that point, one reaches step 6: the tentative full model. With the user’s help to delete ghost atoms, one moves on to step 7: the intermediate partial model. After defining a new *sR*1, the calculation goes back to steps 2, 3, 4, and then repeats step 5 for certain cycles, and then goes back to step 2 again *etc*., until a new tentative solution is produced. If the new tentative solution still has many ghost atoms, the above calculations will again be repeated. Once the new tentative solution has high quality, one reaches step 8: the final model.

To summarize, a general flowchart of applying *sR*1 for solving an unknown structure is shown in Fig. 7 ▸.

## 11. Comparison with other methods

The Patterson deconvolution techniques (Richardson & Jacobson, 1987 ▸; Pavelčík, 1988 ▸, 1994 ▸; Burla *et al.*, 2004 ▸, 2006*a* ▸,*b* ▸, 2007 ▸; Caliandro *et al.*, 2008 ▸) succeed in providing initial phasing for small molecules up to protein structures. However, the Patterson deconvolution techniques typically can only serve an initial phasing purpose since they are not powerful enough to solve a complete structure on their own. In comparison, the *sR*1 method can independently solve a complete structure. This is due to its power of gradually revealing the structure. Starting from an arbitrarily positioned single atom, *sR*1 reveals a little of the structure at the start. Once a little is known, more can be revealed, and then much more can be revealed. In the end, a full structure is revealed. When needed, poorly determined parts of the structure can be deleted and rebuilt. All these abilities have been demonstrated with test calculations of small structures. In the future, we will test how the *sR*1 method can meet the challenges of protein structures.

The diagonal least-squares technique was successful in performing *ab initio* phasing of small molecules (Burla *et al.*, 2018 ▸). Mathematically, the least-squares method requires the starting solution to be close to the correct solution to converge, because it uses Taylor expansion to linearize the model. Therefore, the success of the least-squares technique relies on a multi-solution strategy by trying many initial guesses, combined with its ability to discern which atoms are the bad ones and which therefore need to be replaced by new random guesses. In comparison, the *sR*1 method is deterministic. However, its initial result may contain ghost atoms; thus, it requires a user to identify and delete the ghost atoms and then re-extend back to the full model by resuming the *sR*1 calculations.

The maximization target of the LLGI was successful in locating up to ten S atoms in a protein structure with 2525 non-hydrogen atoms (McCoy *et al.*, 2017 ▸). The theoretical basis for LLGI is statistical hypothesis testing (Read, 2001 ▸), which is theoretically sound and sophisticated, but the formulas of LLGI are complicated, with unknown parameters that need to be estimated and/or optimized. In comparison, the basis of the *sR*1 minimization target is simple mathematical intuition: the correct model has the smallest *R*1 factor. This is the beauty of the *sR*1 target, leading to simple and transparent formulas with no unknown parameters (except for the cell content). It is surprising that such a simple idea can effectively solve crystal structures. One main reason for this success is that the part relating to the effects of the missing atoms *j* + 1 to *N* on the *R*1 factor that is independent of their location, namely, 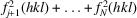 , is retained in equation (5). This part accounts for the baseline effect of these missing atoms on the *R*1 factor. Because the effect of the probe atom *j* on *R*1 is weak, if this baseline effect is not accounted for, the weak effect of the probe would be lost in noise. Though the effect of the probe is weak, its variation with (*x*_*j*_, *y*_*j*_, *z*_*j*_) is consistent such that all missing atoms appear as holes in the *sR*1 map. Just because the missing atoms leave such discernible trace footprints on the *sR*1 map, the *sR*1 method turns out to be successful. (In retrospect, we are still amazed that this simple *sR*1 idea can solve crystal structures, because it employs an oversimplified model: the atomic electron densities are spherically shaped without any thermal effects. There is no way that such an oversimplified model can mimic the real electron density. However, the magic is that, even though the model can never fit the real electron density, it can accurately locate the central portion of the real electron density of each real atom, such that it is good at locating the atoms without fitting the complete electron-density map.)

As far as we can see, the main advantage of the *sR*1 method is its ability to gradually reveal an unknown structure, and the main disadvantage is its slow rate of calculation. Conceptually, the calculation time is proportional to the product of the number of atoms, the number of grid points and the number of reflections. All these numbers are proportional to *N*, the number of atoms in the cell. Thus, roughly speaking, the calculation time is proportional to *N* ^3^. We have not implemented parallel calculation. However, the *sR*1 method is well suited for parallel calculation. For example, the calculation over all grid points can be executed simultaneously. We expect that with parallel programming the *sR*1 method can turn into a usable tool. Up to this point, we have only tested *sR*1 with small molecules and without parallel programming. For sample 3, *sR*1 needs about 1 h to solve a structure with a Python program without parallel calculation on a Surface Pro 7. In comparison, the *SHELXT* program (Sheldrick, 2015 ▸) can solve it in seconds. Clearly, the *sR*1 method, as we have implemented it to this point, has no practical advantages for solving small structures. It can only serve an enrichment purpose: it is viable to solve structures via the *sR*1 method. It is probable that *sR*1 may find practical applications for solving protein structures. However, that area is still awaiting exploration. At this point, it is too early to speculate how the *sR*1 method can meet the challenges of protein structures.

## 12. Future directions

In the current implementation of using *sR*1 to solve a crystal structure, no prior knowledge of the symmetry of the crystal is required. After solving the structure, the crystallographic symmetry can be determined by examining the content of the unit cell (see Section 2). However, when prior knowledge of the symmetry does exist, that fact can be exploited to shorten the calculation time. Considering the crystallographic symmetry (including centering), a group of symmetry-related atoms are assigned together, at the locations of **A**_*i*_ **r** + **d**_*i*_, for *i* = 0 to *n* − 1, where **A**_0_ is the unit matrix and **d**_0_ = (0, 0, 0). The whole group is completely determined by **r** = (*x*, *y*, *z*), thus reducing 3*n* dimensions to three dimensions. Because a group of symmetry-related atoms are assigned together, the concept *pR*1 should be used. The algorithm should watch out for special positions: within the group of the symmetry-related atoms, if two or more atoms are clustering together, namely, their interatomic distance is less than, say, 0.5 Å, these atoms should be replaced by a single atom at their average location. Now **r** = (*x*, *y*, *z*) of the first group of symmetry-related atoms should be determined by the deepest hole of the corresponding *pR*1 map. Afterwards, this group of atoms serve as the starting known atoms. Once there are known atoms, the calculation can be accelerated by using the holes in an *sR*1 map to predict the possible locations of **r** = (*x*, *y*, *z*) for the next batch of symmetry-related atomic groups. Only these predicted locations need to be examined for determination of the deepest *pR*1 hole for locating a group of symmetry-related atoms.

One thing hindering the automation of the *sR*1 calculation is its requirement of a user’s manual help for identifying and deleting ghost atoms. With accumulation of experience of applying the *sR*1 method, in the future such pattern recognition of human intelligence can ultimately be automated. For now, we are testing one automation strategy: let *sR*1 finish the initial steps of building a first trial full model, then repeat the following step: randomly delete half of the model and rebuild back to the full model. The process is monitored by the *R*1 value. Initially, *R*1 drops. Later, it stays at an average level with some ripples. This state signals the end of the calculation. This strategy has been tested and found to be successful for all three examples reported in this paper. More studies will be performed. Alternatively, techniques for identifying ghost atoms can be borrowed from published studies (Kinneging & Graaff, 1984 ▸; Shi & Schenk, 1988 ▸) and will be tried in future explorations.

Finally, the concept of partial structure *R*1 (*pR*1) offers a new way of doing molecular-replacement calculations. The correct orientation of a free-standing fragment, or a partial structure, or sometimes called a partial model, can be determined by the deepest hole in a *pR*1 map of a three-dimensional orientation space. When some atoms of the model have already been determined, the orientation and location of a missing fragment can be determined by the deepest hole in a *pR*1 map of a six-dimensional orientation-location space. After determination of this fragment, a new *pR*1 can be defined by including the atoms of this fragment as known atoms, and the deepest hole of this new *pR*1 map determines the orientation and location of the next missing fragment. In this way, the missing fragments are sequentially determined. In this implementation of *pR*1 for doing molecular-replacement calculations, no prior knowledge of crystallographic symmetry is required. Similarly to using *sR*1, if prior knowledge of crystallographic symmetry does exist, that fact can likewise be exploited to reduce the calculation time. (At the time of this writing, we have tested *pR*1 for molecular-replacement calculations on small structures with success and are planning further testing with protein structures.)

## Supplementary Material

Supporting information. DOI: 10.1107/S2053273324001554/ae5140sup1.pdf

## Figures and Tables

**Table 1:** Crystallographic information of the three samples that are studied in detail in this report

Sample	Formula (excluding H)	*Z*	Non-H atoms in cell	*a* (Å)	*b* (Å)	*c* (Å)	α (°)	β (°)	γ (°)	Space group
1	S_2_O_2_C_12_	2	32	5.86	10.34	10.74	90	104.50	90	*P*2(1)
2	C_46_	1	46	5.95	10.80	12.97	103.77	99.95	90.46	*P* 1
3	C_78_	2	156	12.34	15.98	16.57	114.10	90.70	103.20	*P* 1

**Table 2:** List of the distances between the minimum point of an *sR*1 hole and the location of its corresponding missing atom (unit: Å)

Atoms	*sR*1(S2)	*sR*1(O7)	*sR*1(C20)
S2	0.31		
S3	0.04		
S4	0.30		
O5	0.17		
O6	0.33		
O7	0.05	0.03	
O8	0.16	0.05	
C9	0.00	0.04	
C10	0.09	0.06	
C11	0.25	0.13	
C12	0.12	0.05	
C13	0.41	0.08	
C14	0.24	0.11	
C15	0.27	0.11	
C16	0.13	0.06	
C17	0.18	0.08	
C18	0.34	0.11	
C19	0.31	0.10	
C20	0.29	0.15	0.09
C21	0.15	0.06	0.04
C22	0.25	0.04	0.03
C23	0.24	0.09	0.05
C24	0.33	0.06	0.04
C25	0.36	0.11	0.06
C26	0.00	0.11	0.06
C27	0.07	0.08	0.05
C28	0.21	0.07	0.05
C29	0.36	0.00	0.09
C30	0.10	0.19	0.13
C31	0.31	0.08	0.07
C32	0.24	0.14	0.08
