# Supplementary material for: The single-atom R1: a new optimization method to solve crystal structures
Source: Acta Crystallogr A Found Adv. 2024 Mar 18;80(Pt 3):237–48. doi: 10.1107/S2053273324001554 (PMC11067948; doi:10.1107/S2053273324001554)
Supplement: Supplementary file 1 [file a-80-00237-sup1.pdf]

FOUNDATIONS
ADVANCES

Volume 80 (2024)

Supporting information for article:

The Single-Atom R1: a new optimization method to solve crystal structures

Xiaodong Zhang and James P Donahue

S1. Information on data collection of the samples

All crystals were coated with paratone oil and mounted on the end of a nylon loop attached to the end of the goniometer. Data were collected at 150 K under a dry N_2 stream supplied under the control of an Oxford Cryostream 800 attachment. The data collection instrument was a Bruker D8 Quest Photon 3 diffractometer equipped with a Mo fine-focus sealed tube providing radiation at λ = 0.71073 nm or a Bruker D8 Venture diffractometer operating with a Photon 100 CMOS detector and a Cu Incoatec I microfocus source generating X-rays at λ = 1.54178 nm.

S2. Profiles of a typical sR1 hole

Figure S1 shows typical profiles of an sR1 hole. Along a straight line passing through the minimum point of the hole, the local minimum point of the hole is sandwiched between two neighboring local maximum points, and the distance between these two maximum points is larger than 1.2 Å.

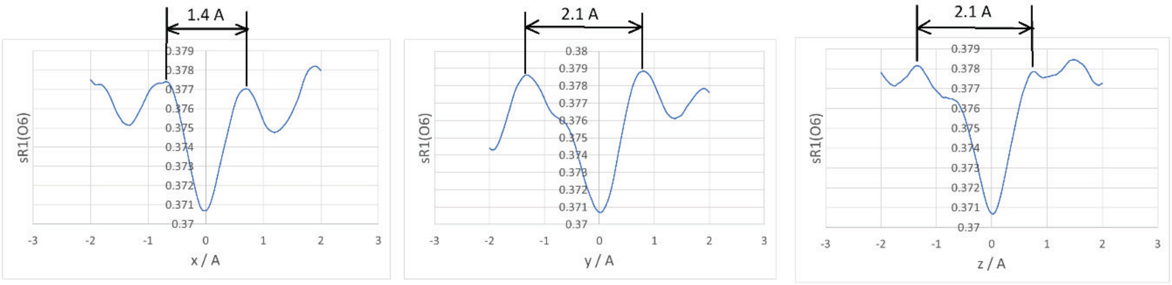

Figure S1 Profiles of sR1(O6) hole next to the missing atom C9. The profiles are calculated along straight lines passing through the minimum point of the hole, along directions of x, y, and z, respectively. The origin is set at the minimum point.

S3. Effect of error in cell content on the structure solution by sR1 method

Correct cell content of sample 1 is S4O4C24. Figure S2 shows the final structure solution by sR1 method when the initial estimate of this cell content is off by about 20%. In one case, cell content is underestimated to be S3O4C19. In this case, the final structure solution has all atoms located correctly, but has some atoms missing. It is possible to guess that one S and five C atoms are missing. In another case, cell content is overestimated to be S5O4C29. In this case, the final structure solution is complete, except that there are six spurious atoms, and one C atom is mis-assigned to be S atom.

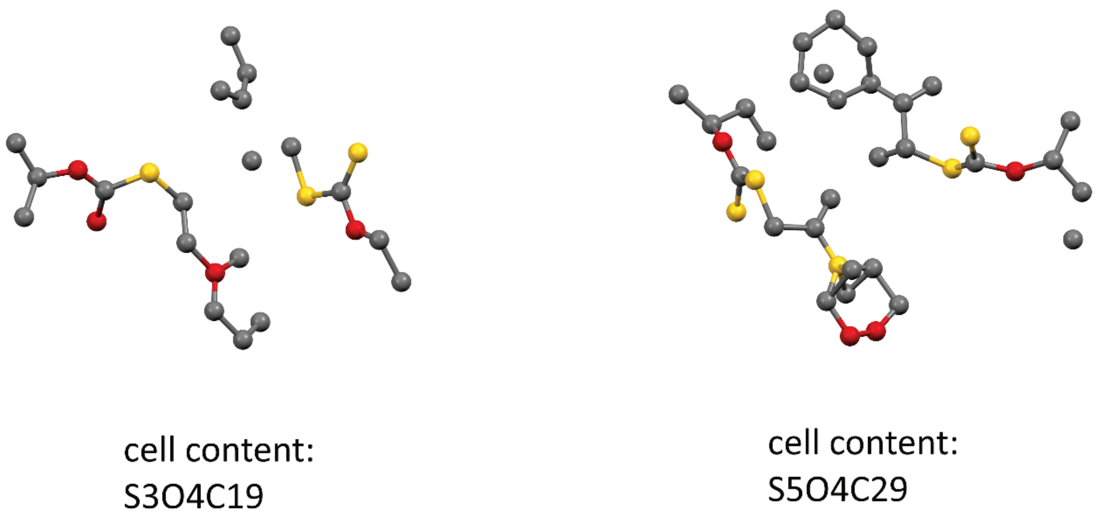

Figure S2 Effect of cell content on the final structure solution of sample 1 by using sR1 method

Correct cell content of sample 2 is C46. Figure S3 shows the final structure solution by sR1 method when the initial estimate of this cell content is off by about 20%. In one case, cell content is underestimated to be C37. In this case, the final structure solution has all atoms located correctly, but has some atoms missing. It is possible to guess that nine C atoms are missing. In another case, cell content is overestimated to be C55. In this case, the final structure solution is complete, except that there are nine spurious atoms, which should be deleted.

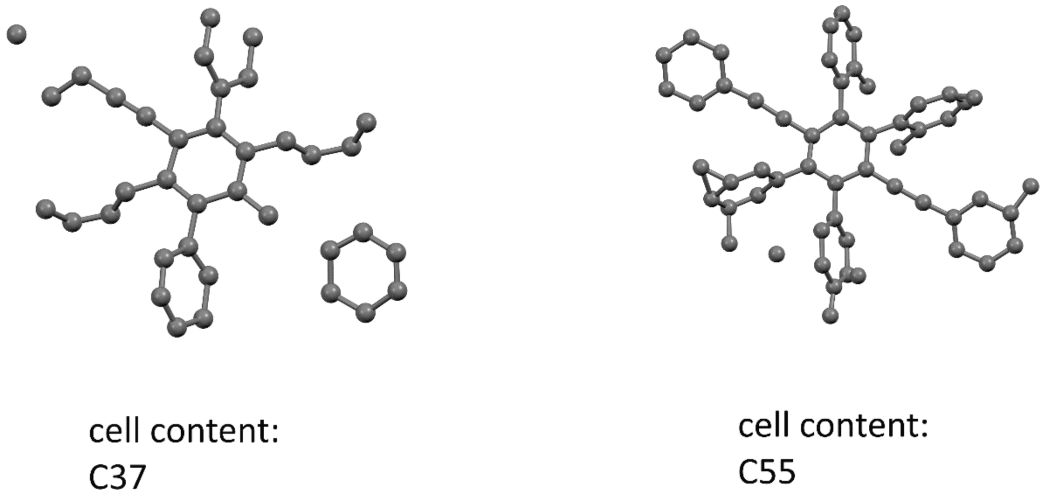

Figure S3 Effect of cell content on the final structure solution of sample 2 by using sR1 method

Correct cell content of sample 3 is C156. Figure S4 shows the final structure solution by sR1 method when the initial estimate of this cell content is off by about 20%. In one case, cell content is underestimated to be C124. In this case, the final structure solution has all atoms located correctly, but has some atoms missing. It is possible to guess that 32 C atoms are missing. In another case, cell content is overestimated to be C188. In this case, the final structure solution is complete, except that there are 32 spurious atoms, which should be deleted.

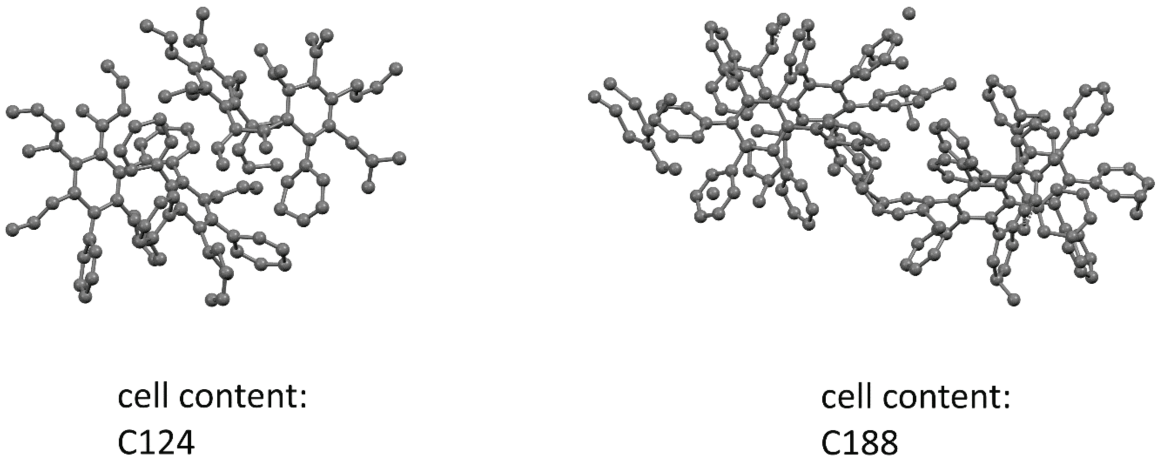

Figure S4 Effect of cell content on the final structure solution of sample 3 by using sR1 method

S4. Effect of error in the scaling of the observed reflection intensities on the performance of the sR1 method

We will compare three scaling schemes: (1) the default scaling, namely the intensities are scaled such that their sum equals $\Sigma_{hkl}\,\Sigma_j\, f_j^2(hkl)$; (2) the doubled scaling, namely after the default scaling the intensities are further multiplied by 2; and (3) the halved scaling, namely after the default scaling the intensities are further divided by 2.

For sample 1 the calculation strategy is: starting from single S atom that is placed at (0.3,0.3,0.3), expand to 2, to 3, to 4, to 12, to 24, and to 32 atoms. If applying the default scaling the resulting model has no ghost atoms:

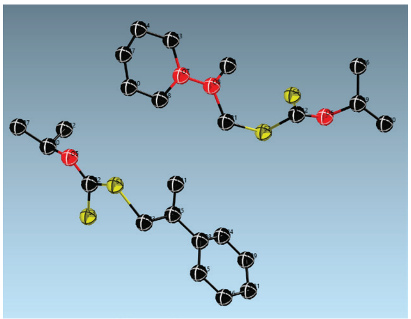

If applying the doubled scaling the resulting model has one ghost atom:

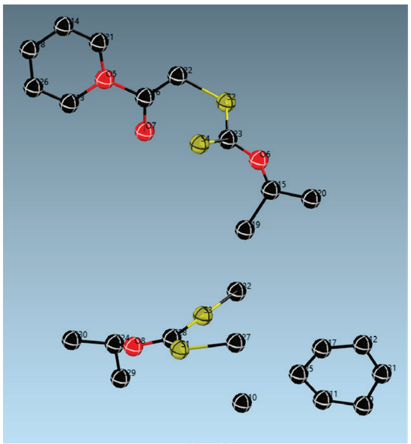

If applying the halved scaling the resulting model has six ghost atoms:

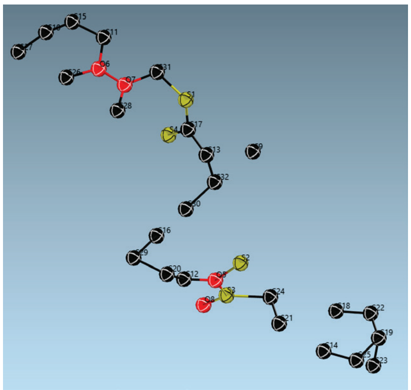

For sample 2 the calculation strategy is: starting from single C atom that is placed at (0.3,0.3,0.3), expand to 5, to 10, to 20, to 33, and to 46 atoms. Using the default scaling the resulting model has no ghost atoms:

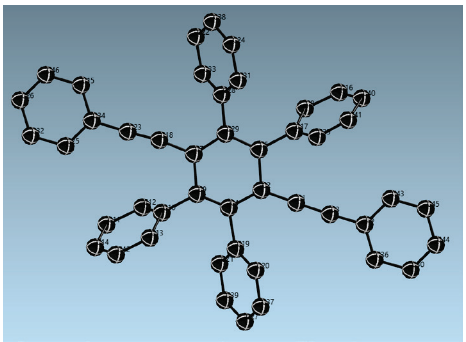

If applying the doubled scaling the resulting model has three ghost atoms:

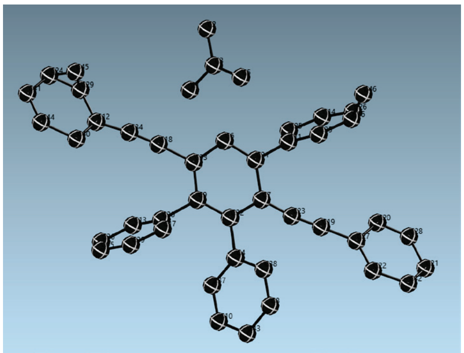

If applying the halved scaling the resulting model has four ghost atoms:

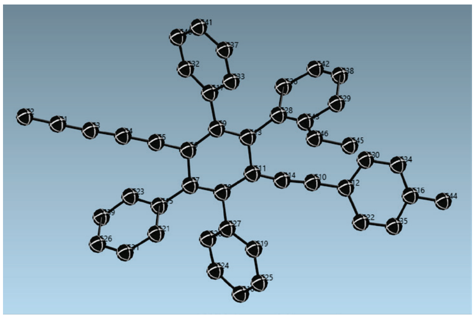

For sample 3 the initial calculation strategy is: starting from a single C atom that is placed at (0.3,0.3,0.3), expand to 10, to 30, to 80, and to 156 atoms. If applying the default scaling the resulting model has five ghost atoms:

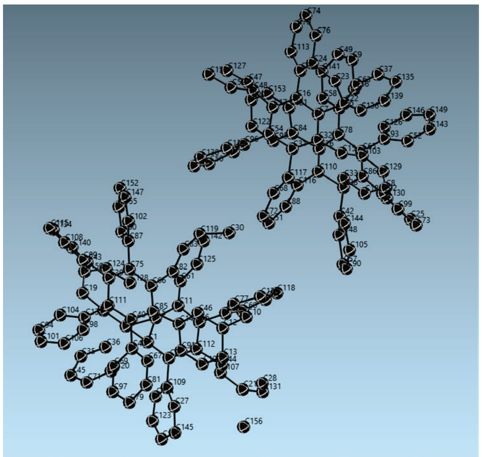

If applying the doubled scaling the initial resulting model is mostly unrecognizable:

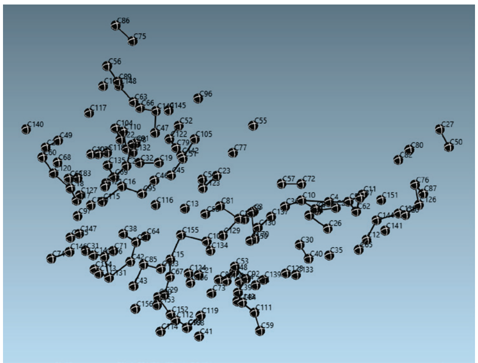

Pick a small fragment from this result and go through the sR1 calculation again. But the new result is still mostly unrecognizable. From the new result pick another fragment and go through the sR1 calculation one more time. This time we recognize the following fragment:

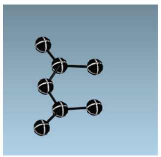

Go through the sR1 calculation another time to improve this fragment to:

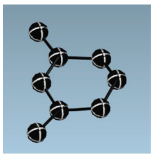

Then go through the sR1 calculation one more time and delete ghost atoms from the result we get the following partial model:

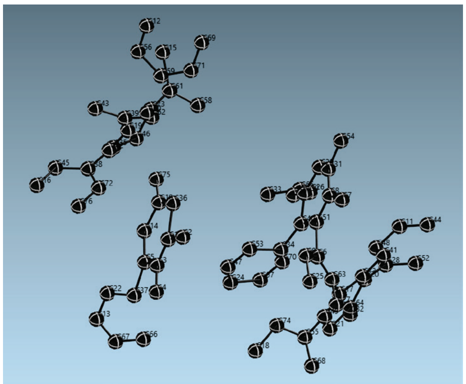

Using the sR1 method to expand back to full model, the final result has four ghost atoms:

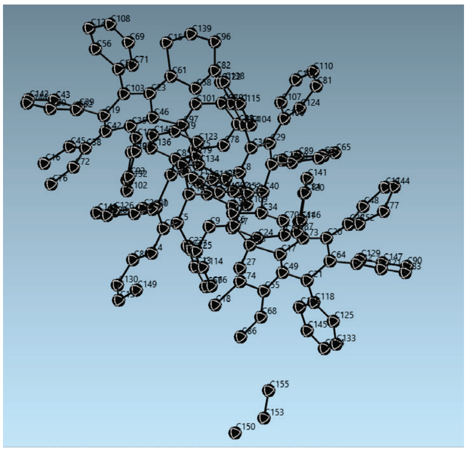

If applying the halved scaling the initial result is the following interesting pattern:

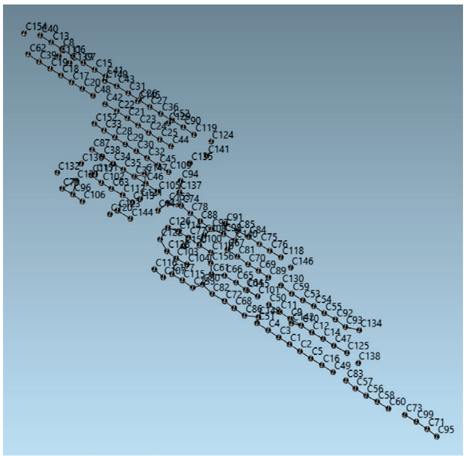

From this pattern we choose the following fragment:

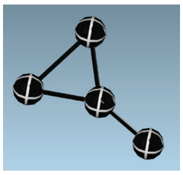

Then go through the sR1 calculation and delete the ghost atoms we reach the following partial model:

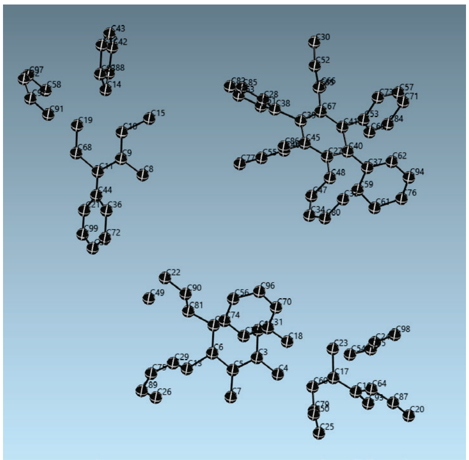

Using the sR1 method to expand back to the full model and delete the ghost atoms we reach the following improved partial model:

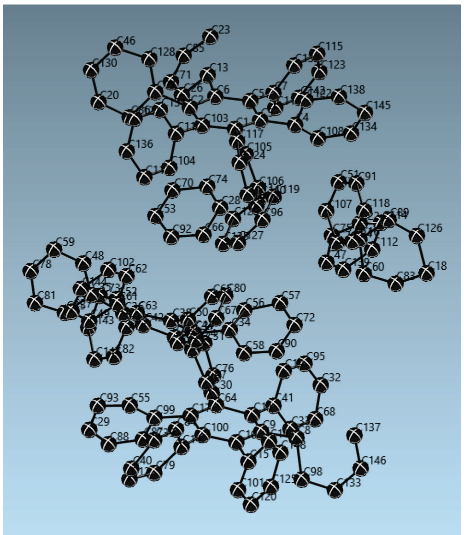

Expanding back to full model one more time, the final result has two ghost atoms:

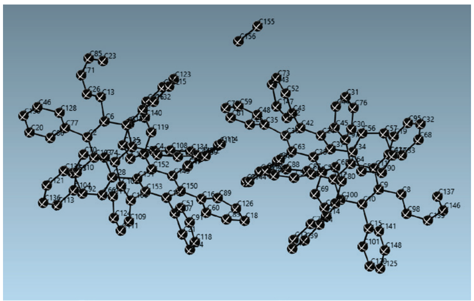

Conclusion: The best scaling scheme is the default scaling, namely, the intensities should be scaled such that their sum equals $\Sigma_{hkl}\,\Sigma_j\,f_j^2(hkl)$. Using the doubled or halved scaling, the sR1 method still works correctly, but the performance is worse in terms of more ghost atoms during the calculation. That means the road towards the final solution will be rough, sometimes may almost lost in totally

unrecognizable results, though user can select small and likely correct fragment to restart the calculation and get back to a correct track.

S5. Technical details of locating global minimum point of an sR1

To locate the global minimum point of an sR1, a coarse grid of 0.4 Å step size is set within the unit cell, as well as one step outside each edge of the cell. sR1 value is calculated on all grid points. The grid point with the lowest sR1 value is selected as the global minimum point. The precision of this minimum point is further refined by setting up a local grid with halved step size. This local grid is centered at previously set location of the minimum point, with one step up and one step down along each direction of x, y, and z. So, the local grid contains 3 by 3 by 3 total 27 grid points. sR1 are calculated over all 27 grid points. The grid point with lowest sR1 is set as the improved location of the minimum point. This step is repeated until the step size is smaller than 0.001 Å, to reach the final answer of the global minimum point of sR1.

S6. Technical details of locating all sR1 holes within a unit cell

To locate all sR1 holes within a unit cell, a coarse grid of 0.4 Å is set within the unit cell, as well as one step outside each edge of the cell. sR1 values are calculated on all grid points. All grid points within the unit cell are tested: a grid point marks a hole if all its six neighbours have higher sR1 values. Precision of locating a hole is further refined by setting up a local grid of halved step size, centered at the previous assigned location of the hole, with one step up and one step down along each direction of x, y, and z. So, this local grid has 3 by 3 by 3 total 27 grid points. sR1 values are calculated over all 27 grid points, and the grid point with the lowest sR1 value is taken as the improved location of the hole. This step can be repeated to reach higher precision. For purpose of locating all hole within the unit cell, precision is only improved to 0.1 Å. Once a hole is identified as the deepest hole, the precision is further improved to 0.001 Å.

S7. Technical details of determination of the correct orientation of a known fragment by global minimum point of the pR1 of a free-standing fragment

A local Cartesian coordinate system is set up for the fragment. The origin of the system sits at the average (weighted by the number of electrons) location of all the atoms in a fragment. The orientation of the system is defined by three unit vectors **x**, **y**, and **z**. To define these unit vectors, two reference vectors **r** and **s** are selected. A reference vector is defined by two reference points: a reference vector is a vector from reference point A to reference point B. The location of an atom, or any other convenient point can be used as a reference point. The unit vectors **x**, **y**, and **z** are related to the reference vectors **r** and **s** as follows: $\mathbf{x} = \mathbf{r}/|\mathbf{r}|$, $\mathbf{y} = (\mathbf{s}\text{-}\mathbf{s}\cdot\mathbf{x}\mathbf{x})/|\ \mathbf{s}\text{-}\mathbf{s}\cdot\mathbf{x}\mathbf{x}\ |$, $\mathbf{z} = \mathbf{x}\times\mathbf{y}$.

For benzene ring, the vector from its center to one C atom is selected as reference vector **r**, while the vector from the center to one neighbouring C atom is selected as reference vector **s**. However, the relations between the unit vectors **x**, **y**, and **z** and the reference vectors **r** and **s** are taken as: **y** = **r**/|**r**|, **z** = (**s**-**s**·**yy**)/| **s**-**s**·**yy** |, **x** = **y**×**z**. In this way, the 6-fold symmetry axis is along the x-axis.

A Cartesian coordinate system is also set up for the unit cell. The origin of this system overlaps the origin of the unit cell. Its three unit vectors **x**, **y**, and **z** are related to the unit cell vectors **a** and **b** as follows: **x** = **a**/|**a**|, **y** = (**b**-**b**·**xx**)/| **b**-**b**·**xx** |, **z** = **x**×**y**. The Cartesian coordinates of this system and the fractional coordinates of the unit cell are inter-converted during the calculations. When doing translational calculations a grid of 0.4 Å step size is set up within the unit cell.

At start, a fragment is located such that its local Cartesian system overlaps the Cartesian system of the unit cell. The fragment is attached to its local Cartesian system. So, rotation and translation of the fragment is realized by rotating and translating its local Cartesian system in the cell Cartesian system. Translation is realized by translating the local origin in the cell Cartesian system (or rather in the cell fractional coordinate system, which is converted to the cell Cartesian system). A general rotation is consisted by three simple rotations: a rotation around x-axis (of the local frame) in the direction from y-axis to z-axis through angle ψ; a rotation around z-axis (of the local frame) in the direction from x-axis to y-axis through angle φ; and another rotation around x-axis (of the local frame) in the direction from y-axis to z-axis through angle ζ.

The space spanned by rotation and translation is a 6-dimensional orientation-location space. (It is 5-dimensional if the fragment is linear.) To coarsely locate the global minimum point or the holes of a pR1 map in this 6-dimensional space, a coarse grid is set up with 0.4 Å step size in translations within the cell and 5-degree step size in rotation angles. The range of rotation angles are: 0 to 360 degrees for ψ, 0 to 180 degrees for φ, and 0 to 360 degrees for ζ. If the fragment has n-fold rotation symmetry, and the rotation axis is arranged along the local x-axis, the range of ζ can reduce to 0 to 360/n. The precision of locating the global minimum point or the local minimum points is refined by halving the step size locally five times.

For a free-standing fragment, that is, a model consisting of a single fragment with no other known atoms, the pR1 only depends on the orientation of the fragment. Therefore, the possible orientations of all missing fragments are detected by the holes in the pR1 map of a free-standing fragment in a 3-dimensional orientation space. Due to possible symmetry of a fragment and the redundancy in representing an orientation with the three rotation angles, many orientation representations are equivalent to each other. Considering two orientation representations, if the atoms of the fragment of one orientation representation can match the atoms of the fragment of the other orientation representation in a one-to-one basis within 0.5 Å, the two representations are considered equivalent. (In this comparison, the types of atoms are ignored. This practice is fine with most fragments.

However, in rare cases the type of atoms should be considered, and the algorithm need to be adjusted, with a penalty of longer calculation time.) The non-equivalent orientation representations are filtered out from all detected representations. These serve as the candidate orientations.

The candidate orientations are ranked from smallest R1 to highest R1 and are labelled by 0, 1, 2, etc. To determine one fragment with correct orientation, the candidate orientation 0 is used.

S8. A few other examples that have been tested with the sR1 method

In the body of this report, we have only selected three examples. We have only included three examples so that we can provide an in-depth analysis. However, we have tested the method over about 50 data sets. Here are a few other examples.

This example has 128 C atoms in the unit cell, which readily yields to the sR1 method:

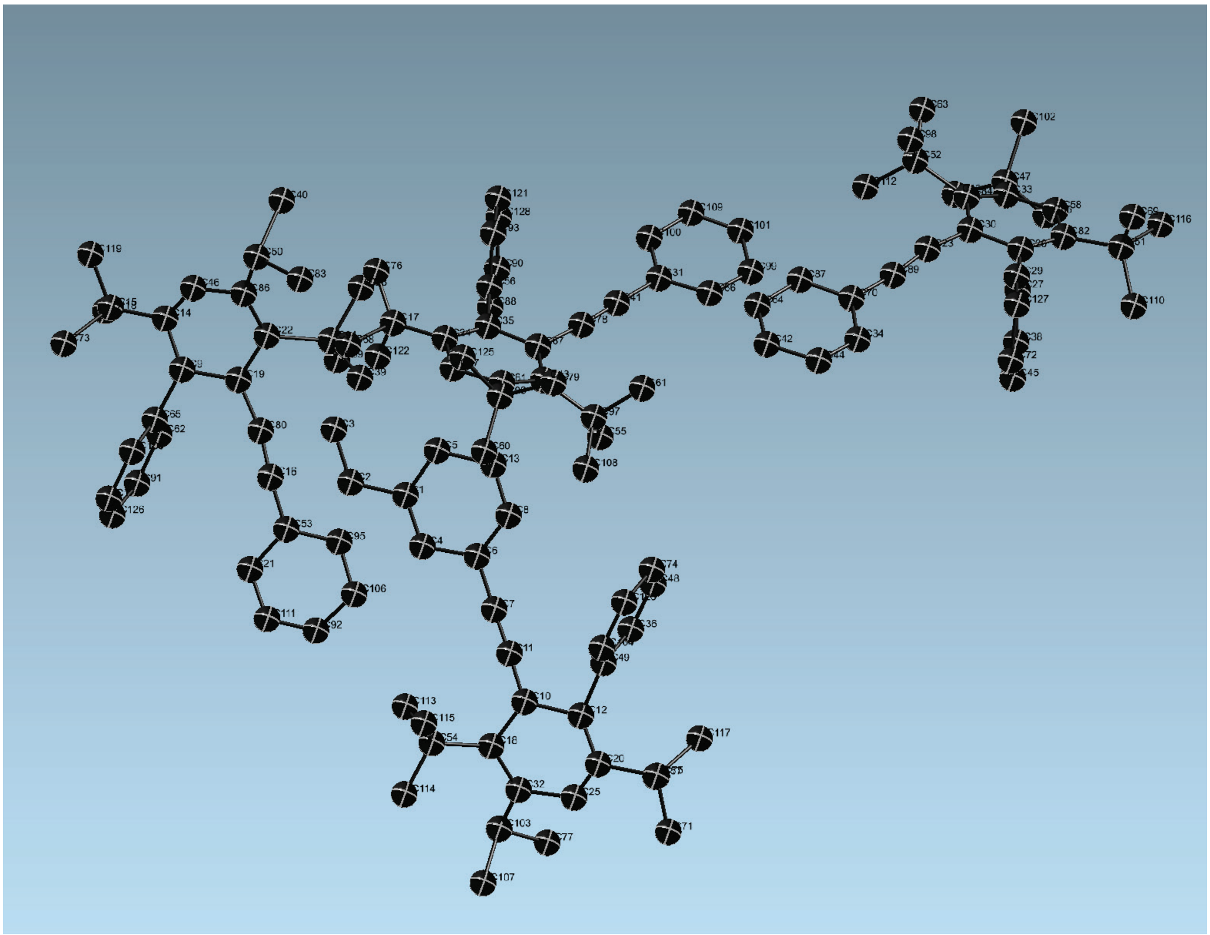

This example has 256 atoms in the unit cell, and the sR1 method can reach the following solution without the user's help of deleting the ghost atoms:

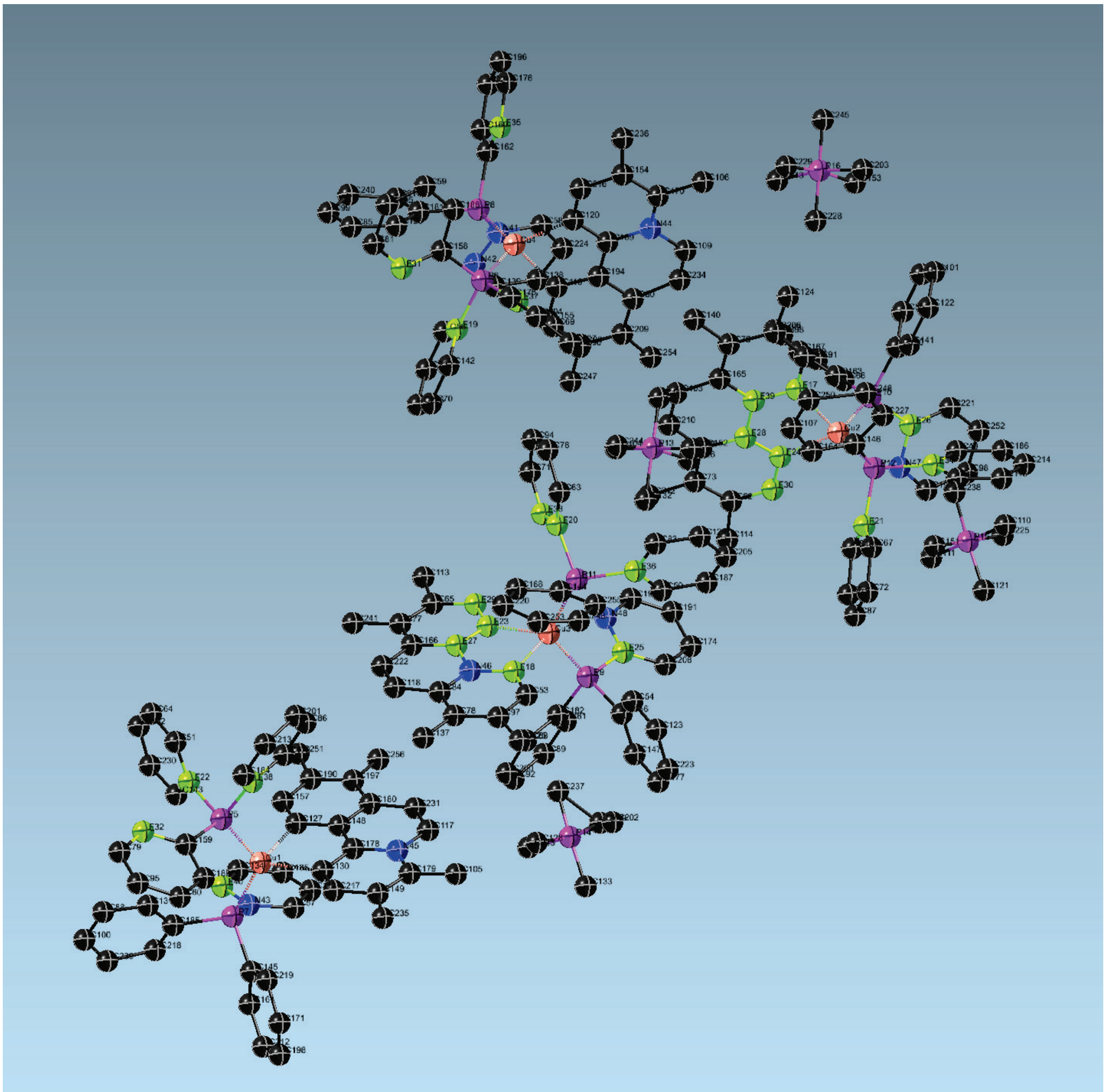

This example has 472 atoms in the unit cell, and the sR1 method can reach the following solution also without the user's help of deleting the ghost atoms:

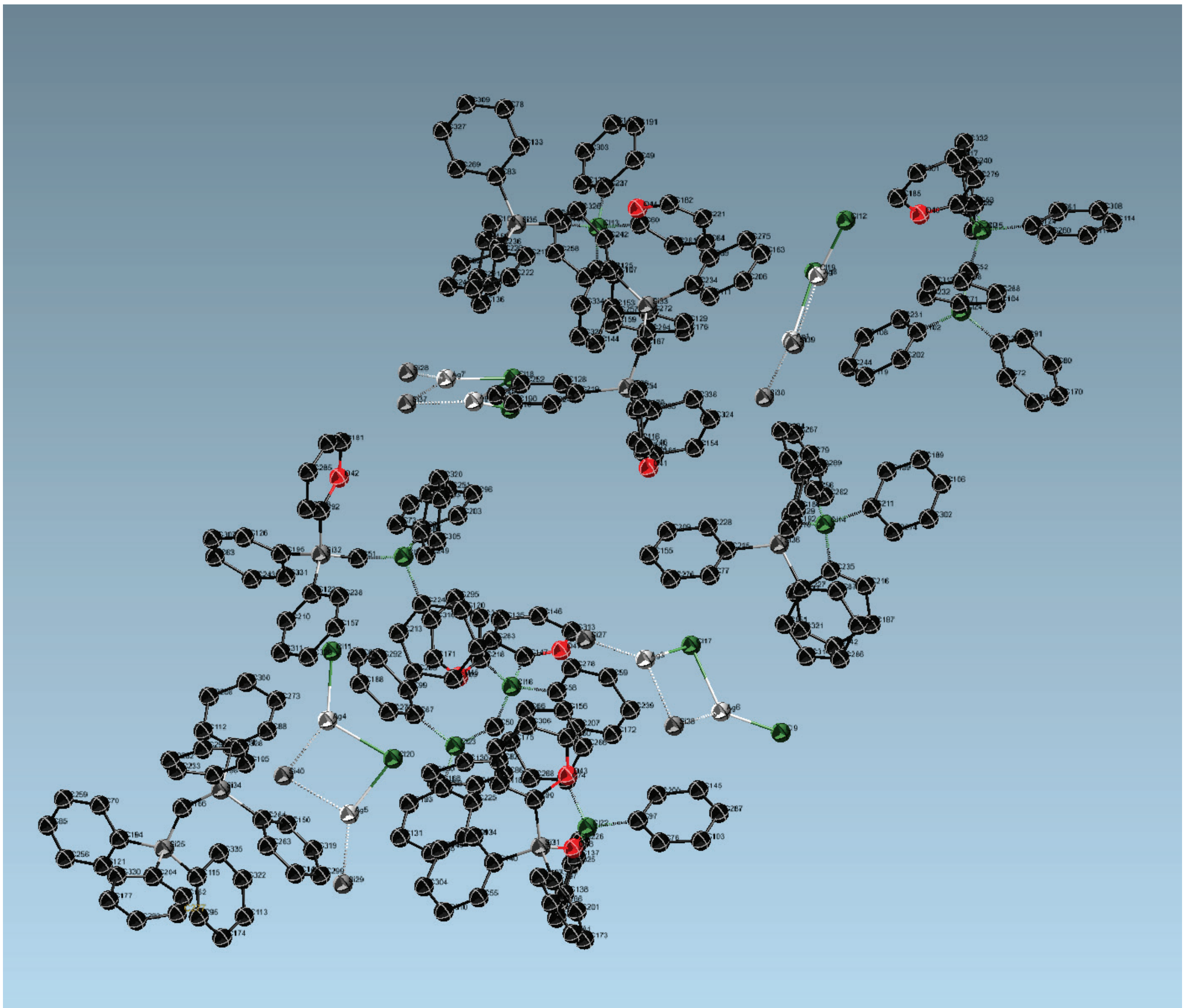

Finally, this example has 696 atoms in the unit cell. The initial solution (without manual editing) is:

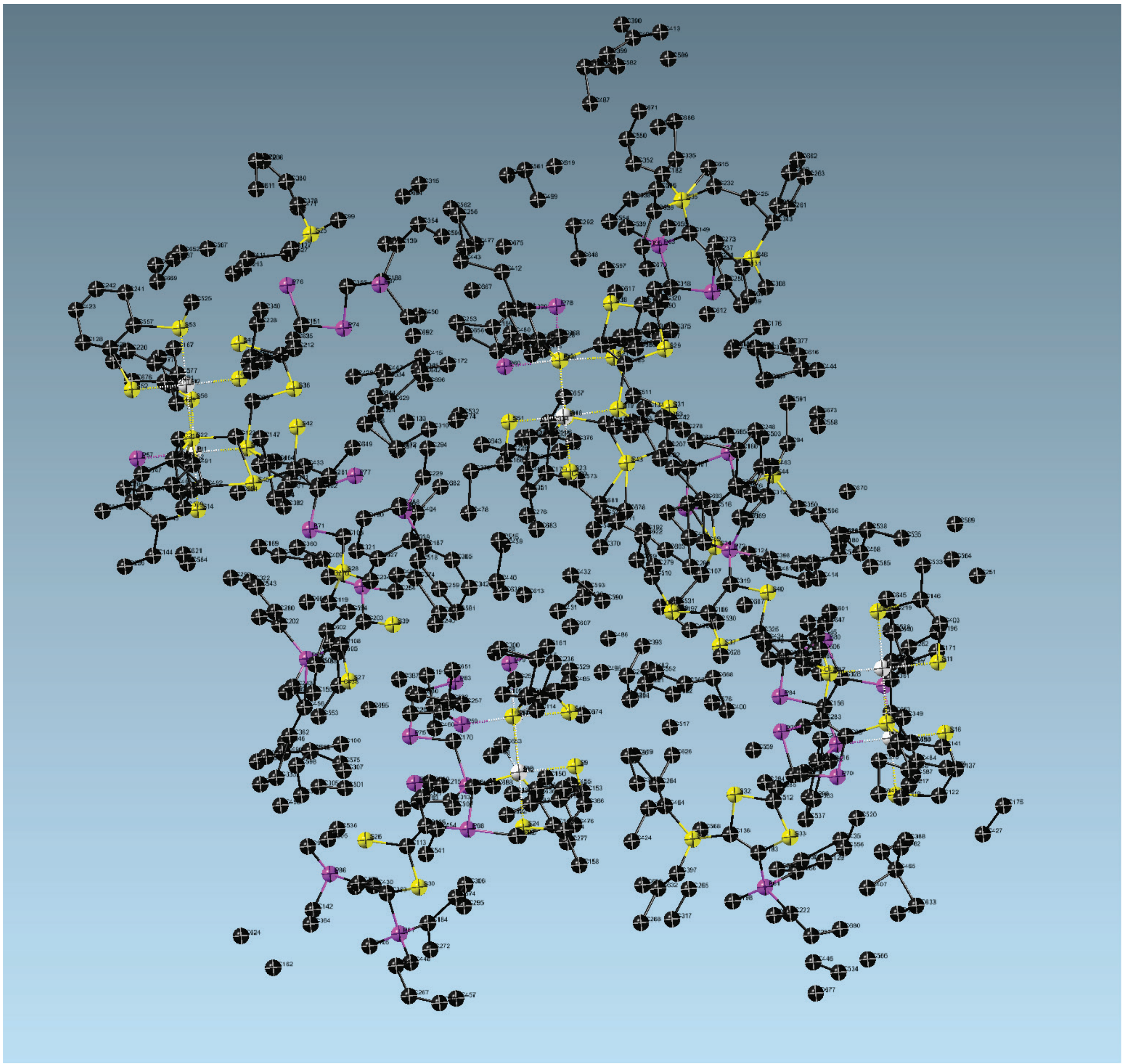

After deleting ghost atoms, use the sR1 method to expand back to full model, and then delete the ghost atoms again, we reach following partial model:

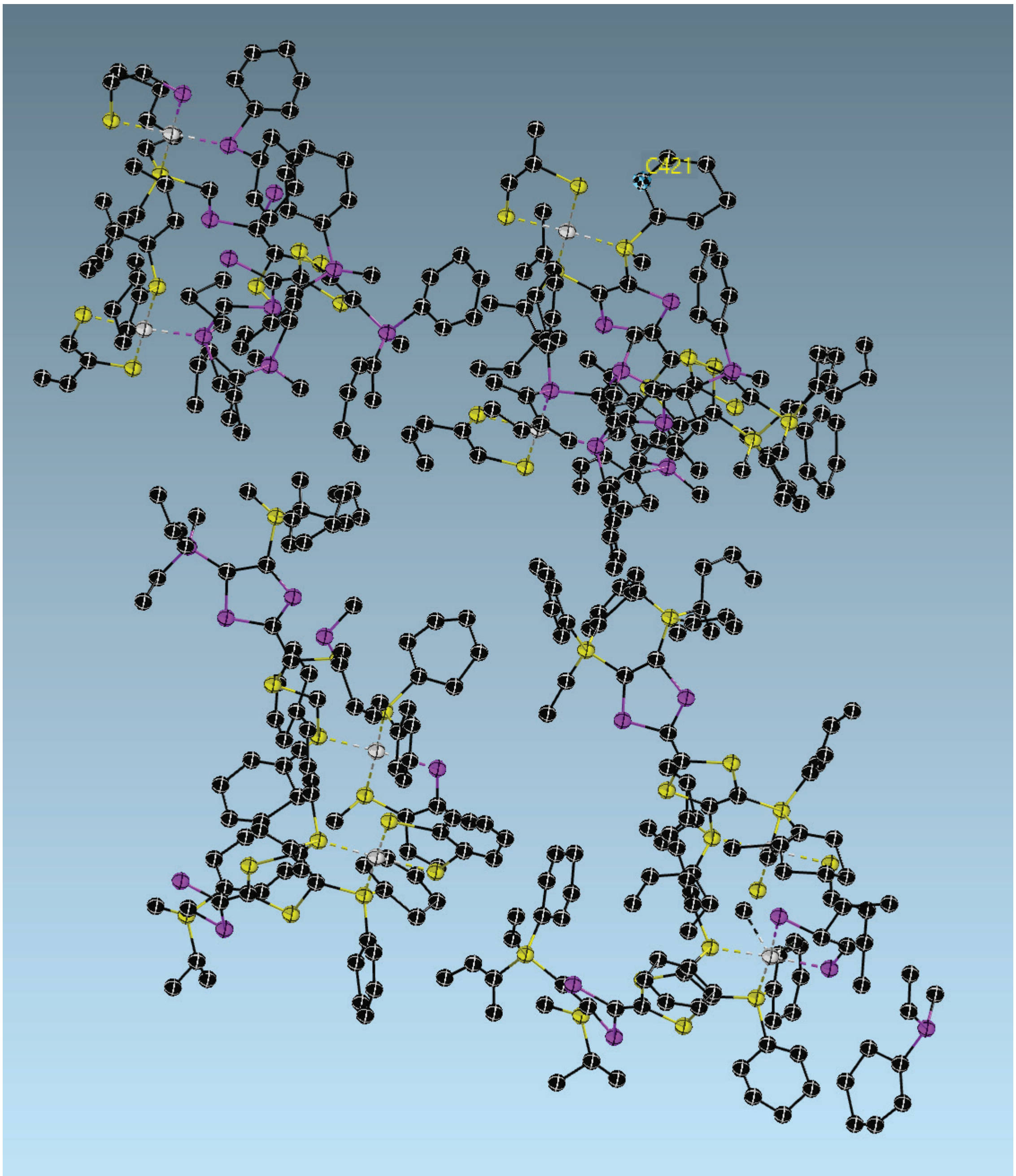

However, further calculations do not improve this partial model substantially.
